# Dynamic neural circuit disruptions associated with antisocial behaviors

**DOI:** 10.1002/hbm.25225

**Published:** 2020-10-16

**Authors:** Weixiong Jiang, Han Zhang, Ling‐Li Zeng, Hui Shen, Jian Qin, Kim‐Han Thung, Pew‐Thian Yap, Huasheng Liu, Dewen Hu, Wei Wang, Dinggang Shen

**Affiliations:** ^1^ Department of Radiology and BRIC University of North Carolina at Chapel Hill Chapel Hill North Carolina USA; ^2^ Department of Information Science and Engineering Hunan First Normal University Changsha Hunan China; ^3^ College of Intelligence Science and Technology National University of Defense Technology Changsha Hunan China; ^4^ Department of Radiology, The Third Xiangya Hospital Central South University Changsha Hunan China; ^5^ Department of Artificial Intelligence Korea University Seoul South Korea

**Keywords:** antisocial behavior, brain network, cognitive control function, default mode network, dynamic functional connectivity, functional MRI, resting state

## Abstract

Antisocial behavior (ASB) is believed to have neural substrates; however, the association between ASB and functional brain networks remains unclear. The temporal variability of the functional connectivity (or dynamic FC) derived from resting‐state functional MRI has been suggested as a useful metric for studying abnormal behaviors including ASB. This is the first study using low‐frequency fluctuations of the dynamic FC to unravel potential system‐level neural correlates with ASB. Specifically, we individually associated the dynamic FC patterns with the ASB scores (measured by Antisocial Process Screening Device) of the male offenders (age: 23.29 ± 3.36 years) based on machine learning. Results showed that the dynamic FCs were associated with individual ASB scores. Moreover, we found that it was mainly the inter‐network dynamic FCs that were negatively associated with the ASB severity. Three major high‐order cognitive functional networks and the sensorimotor network were found to be more associated with ASB. We further found that impaired behavior in the ASB subjects was mainly associated with decreased FC dynamics in these networks, which may explain why ASB subjects usually have impaired executive control and emotional processing functions. Our study shows that temporal variation of the FC could be a promising tool for ASB assessment, treatment, and prevention.

## Graphical abstract

Dynamic functional connectivity (dFC) was shown to be informative in brain‐behavior association. Temporal variability of the inter‐network dFC may contribute to the ASB severity association. Impaired adaptive task control and emotion process could be associated with antisocial behaviors.

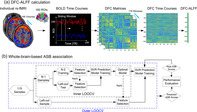

## 1 INTRODUCTION

Antisocial behavior (ASB) is an important public health concern due to its serious societal cost, not only involving directly financial loss but also intangible mental pain and injury. There is increasing interest in research on the neural associations of ASB and the risk prediction for future ASB (Poldrack et al., 2017). Numerous studies in the field of behavioral research have provided evidence that indicates disruptions in cognitive functions in ASB subjects (Klapwijk, van den Bos, & Güroğlu, 2017). These cognitive function disruptions include difficulties in behavioral control (Murray, Waller, & Hyde, 2018), increased impulsivity (Mackey et al., 2017), risk‐taking decisions (Syngelaki, Moore, Savage, Fairchild, & Van Goozen, 2009), and excessive reward‐driven behavior (Murray, Shaw, Forbes, & Hyde, 2017), among others.

Neuropsychological deficits in the cognitive functions are thought to contribute to antisocial and aggressive behaviors according to in vivo brain research using structural and functional magnetic resonance imaging (MRI; Ogilvie, Stewart, Chan, & Shum, 2011). Increasing studies using structural MRI have suggested that ASB might be associated with structural or morphometric abnormalities in the high‐order cognitive function‐related regions. For example, Raine, Lencz, Bihrle, LaCasse, and Colletti (2000) found that individuals with severe ASB had reduced gray matter volume in the dorsolateral and medial prefrontal cortices, further confirmed by their subsequent study (Raine, Yang, Narr, & Toga, 2011). Narayan et al. (2007) found medial frontal cortex thinning in violent ASB individuals. A large cohort study of ASB (*N* = 1,830) revealed that a higher impulsivity level was associated with decreased gray matter volume in the mediofrontal cortex (Mackey et al., 2017). Sundram et al. (2012) used diffusion‐weighted imaging (DWI) and found abnormalities of the fibers in the frontal lobe in ASB. While informative, the fact that ASB subjects usually have impaired behavioral control calls for more functional studies.

The functional abnormalities in the high‐level cognitive function‐related areas were also found in ASB subjects during task‐based functional MRI (fMRI) while the subjects were involved in decision making and reward‐based learning tasks (Klapwijk et al., 2017; Mackey et al., 2017; Murray et al., 2017). For instance, during a monetary reward/loss task, Murray et al. (2017) found that ASB was related to less activation of the ventral striatum during reward anticipation and less activation of the ventrolateral prefrontal cortex during reward and loss anticipation. During decision making with morally distressful stimuli, reduced activations were consistently found in the medial prefrontal cortex, posterior cingulate cortex, amygdala, and anterior temporal areas in individuals with severe ASB (Harenski, Harenski, Shane, & Kiehl, 2010; Marsh & Cardinale, 2014; Pujol et al., 2012). Recently, to investigate brain function in more natural circumstances and thus rule out potential confounding factors raised by the task executions, our group has been using resting‐state fMRI (rs‐fMRI) to investigate abnormal synchronizations of the spontaneous neural activities among different brain regions with functional connectivity (FC) metrics in subjects with severe ASB, that is, antisocial personality disorders (Tang, Jiang, Liao, Wang, & Luo, 2014). Similar to our findings, Yoder, Harenski, Kiehl, and Decety (2015) suggested that executive function‐related networks could be the most targeted brain functional system in ASB individuals.

Despite the progress in understanding of ASB, previous research was mainly based on group‐level analysis with statistical analysis, and most of the work focused on subjects with severe ASB, for example, those with violent behaviors (Poldrack et al., 2017), antisocial personality disorders (Jiang et al., 2016; Jiang et al., 2017; Tang et al., 2014), and psychopathy (Blair, 2012; Ly et al., 2012). Despite these studies focused on contrasting severe ASB with normal controls, few studies investigated how the brain structure or function changes with different ASB severity levels, that is, the association between brain and ASB scores. Specifically, traditional rs‐fMRI‐based FC studies of ASB usually calculated a single FC matrix or map with the entire rs‐fMRI scan (Contreras‐Rodríguez et al., 2015; Tang et al., 2014). The resultant FC metrics have been interpreted as stable FC or time‐invariant (Lurie et al., 2018; Fan et al., 2008; Fan et al., 2007) which is now considered to reflect “core connections” in the brain functional networks and could not be used to characterize adaptive, complex task control‐related, “dynamic” brain functional organizations (Hutchison et al., 2013; Liu, Zhang, Chang, & Duyn, 2018). Therefore, it is less likely to identify any abnormalities based on such stable FC metrics from the subjects with different ASB severity levels.

To reveal such subtle brain functional changes, dynamic FC (dFC) studies have been recently proposed (Hutchison & Morton, 2016; Medaglia et al., 2018). For example, the temporal variability of the dFC has been found quite informative in detecting the switch of cognitive states (Allen et al., 2014; Lurie et al., 2018) and sensitive to many brain diseases (Jie, Liu, & Shen, 2018; Li, Zang, & Zhang, 2015; Liao et al., 2014; Liu et al., 2018; Suk, Wee, Lee, & Shen, 2016; Yan, Zhang, Sui, & Shen, 2018; Zhang, Chen, Zhang, & Shen, 2017). Hutchison, Womelsdorf, Gati, Everling, and Menon (2013) suggested that dFC may reflect an intrinsic brain property with a neural origin and could index changes in large‐scale neural activity patterns underlying cognition and behavior. Preti, Bolton, and Van De Ville (2017) provided a comprehensive description of the state‐of‐the‐art dFC analysis approaches, including a widely adopted sliding window‐based correlation analysis for capturing time‐resolved FC. By descriptive FC measures over subsequent time windows, fluctuations in FC can be captured (Preti et al., 2017). Currently, investigating temporal variability of dFC is believed as an effective method to characterize FCs' intrinsic temporal fluctuations and to associate functional networks' activities and behaviors (Thompson et al., 2013). In their seminal works, Kucyi et al. used standard deviation of a dFC time course to track mind wandering (Kucyi, Salomons, & Davis, 2013) and daydreaming (Kucyi & Davis, 2014). However, the standard deviation of the dFC measures their temporal variability within the full frequency range. To better remove spurious fluctuations and keep those originated from neural activity and involved in changes in cognitive states or brain diseases (Betti et al., 2013; Leonardi & Van De Ville, 2015; Thompson et al., 2013; Wilson et al., 2015), Shen et al. (2016) measured only low‐frequency component of the dFC time series (dFC‐ALFF, amplitude of the low‐frequency fluctuations of dFC) for investigating taxi drivers' vigilance network and its experience‐related changes. Using dFC‐ALFF to characterize ASB‐related brain functional changes is suitable to better understand the neural associations of ASB.

In this study, we focused on ASB subjects with different severity (including mild and severe ASB, measured by a continuous behavioral scale, Antisocial Process Screening Device [APSD]) and tried to establish the individual‐level associations between dFC‐ALFF and the APSD scores. Specifically, we adopted a recently established machine learning method, namely connectome‐based predictive modeling (CPM; Finn et al., 2015; Fong et al., 2019; Scheinost et al., 2019), to the dFC‐based individualized ASB association analysis. Based on the literature, we have the following hypotheses: (a) dFC‐ALFF can be associated with ASB severity levels, or the ASB score of a single subject can be reflected by his/her dFC pattern; (b) ASB severity could be mediated by the deteriorated dynamic interactions among the high‐level cognitive function‐related brain regions; and (c) There exist specific functional networks (mostly high‐order cognition‐related) that might be more probably targeted by ASB than others.

## 2 MATERIALS AND METHODS

### 2.1 Participants and antisocial behavior measure

A hundred and fifteen native Chinese male participants (age range 18–26 years old, 23.29 ± 3.36) were recruited from the School for Youth Offender of Hunan Province. They were sentenced to receive an “enclosed‐style” reformatory education. Based on their self‐reporting, none of them was excluded due to any brain injury history or any major mental illness, such as schizophrenia, depression, or anxiety neurosis. All the subjects were right‐handed with normal intelligence quotient (IQ, 80–120) according to the Wechsler Adult Intelligence Scale (Table 1) and without any history of substance abuse.

**TABLE 1:** Demographic characteristics of the subjects in this study

Variables	Mean	SD
Gender	Male	
Number	115	
Age (years)	23.29	3.36
IQ	94.28	12.47
APSD score	16.68	5.03

Abbreviations: APSD, Antisocial Process Screening Device (for measuring antisocial behavior severity); IQ, intelligence quotient; SD, standard deviation.

Two senior psychiatrists assessed the ASB severity for all the subjects. The antisocial behavior and psychopathic traits were quantitatively estimated using the APSD, a 20‐item questionnaire based on the Psychopathy Checklist‐revised (PCL‐R; Frick & Hare, 2001; Goodwin, Sellbom, & Salekin, 2015). APSD measures antisocial behavior and psychopathic traits with regards to impulsivity, narcissism, and callousness‐unemotionality, which has been extended to younger adults (Goodwin et al., 2015). Recent studies have shown that APSD is reliable and valid for subjects from different countries (de Wied, van der Baan, Raaijmakers, de Ruiter, & Meeus, 2014; Goulter, Kimonis, & Heller, 2018; Li, Chan, Ang, & Huan, 2017; Pechorro, Maroco, Poiares, & Vieira, 2013), including China (Wang, Deng, Armour, Bi, & Zeng, 2015). The total APSD score reflects the severity of ASB and was used as the target in our study, which ranged from 6 to 29, with a mean of 16.69 and a median of 17 (Figure S1).

In this experiment, we also screened individual emotional status using the Self‐rating Anxiety Scale (SAS; Zung, 1971), which holds good psychometric credentials (Tanaka‐Matsumi & Kameoka, 1986). The SAS has been extensively used in the research of emotional disorders (Dunstan & Scott, 2018). Our subjects had the SAS scores ranging from 24 to 57, with a mean of 37.41 and a median of 36 (Figure S2A).

None of the subjects had access to alcohol for at least 6 months before the MRI scan. Each of them was chaperoned by three regulators while going out for the MRI scan. This study was approved by the Ethical Committee of the School for Youth Offender of Hunan Province and by the Ethical Committee of the Third Xiangya Hospital of Central South University. Written informed consent was obtained from each subject.

### 2.2 Data acquisitions and preprocessing

All rs‐fMRI data were acquired on a 3.0‐T Philips scanner at the Third Xiangya Hospital of Central South University. Before the scan, subjects were instructed to relax, close their eyes, remain awake, and refrain from any specific thinking. After the scan, a brief survey was carried out to confirm that they did not fall asleep during the scan. The rs‐fMRI scans were performed using a gradient‐echo echo‐planar imaging (EPI) sequence with the following parameters: repetition time (TR) = 2,000 ms, echo time (TE) = 30 ms, flip angle (FA) = 90°, slice thickness = 4 mm without gap, number of slices = 36, field of view (FOV) = 128 mm × 128 mm, and matrix size = 64 × 64. The scan lasted for 400 s, during which 200 volumes were obtained.

The rs‐fMRI data were preprocessed based on an in‐house resting‐state BOLD fMRI preprocessing pipeline as used by previous studies (Shen et al., 2016; Zeng et al., 2014, 2018) based on SPM12 (www.fil.ion.ucl.ac.uk/spm). The first five volumes of the scanned data were removed in consideration of the magnetic saturation, and the remaining volumes were slice timing‐corrected and head motion‐corrected. The corrected volumes were normalized to the standard EPI template in the Montreal Neurological Institute (MNI) space and resampled to 3 × 3 × 3 mm^3^ voxels (Luan, Qi, Xue, Chen, & Shen, 2008; Wu, Qi, & Shen, 2006). Spatial smoothing and temporal filtering were performed using a Gaussian filter of 8‐mm full‐width half‐maximum kernel and a Chebyshev band‐pass filter (0.01–0.08 Hz), respectively. Linear detrending was carried out to remove any signal drift. Both filtering and detrending were conducted in a voxel‐wise manner, including the voxels in the white matter and cerebrospinal fluid; therefore, the covariates used in the later step were also filtered and detrended in the same way. Finally, potential influences of physiological noise and artifacts were further reduced by regressing out the nuisance covariates, including six head motion parameters and the mean signals from the white matter, cerebrospinal fluid, and the whole brain, as well as their first‐order derivatives (Chen et al., 2017).

### 2.3 Calculation of dynamic FC and its temporal variability

**FIGURE 1:**
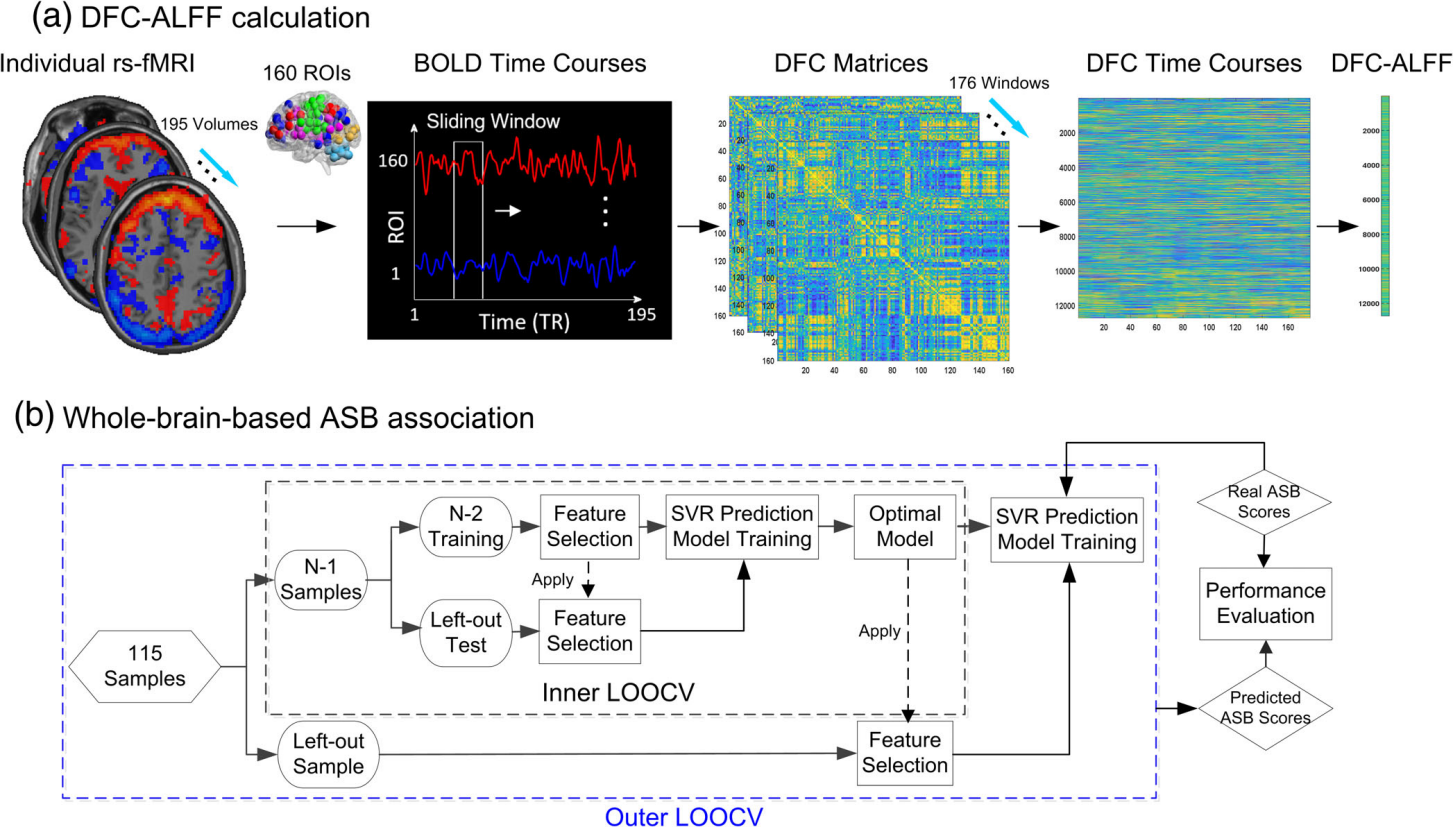
Association analysis of antisocial behavior (ASB) scores with dFC patterns. (a) Individual dynamic functional connections and their variability calculations; (b) Schematic of SVR association with nested leaveone‐out cross‐validation (LOOCV) which consisted of inner and outer layers. The inner LOOCV was used to optimize the predictive model by feature selection (*p*‐value from a predefined range .005–.05, with a step of .001), then the optimal model (the selected features with the optimized *p*‐value) was used to generate the result for the left‐out sample in an outer LOOCV

The processing flowchart is plotted in Figure 1. First, we segmented the preprocessed rs‐fMRI data into 160 regions of interest (ROIs) by using a predefined atlas (Dosenbach et al., 2010). The regional rs‐fMRI signal of each ROI was calculated by averaging the blood‐oxygen‐level‐dependent (BOLD) signals across all voxels in it. A sliding temporal window with a size of 40 s (20 TRs) was slid with a step size of 2 s (1 TR) along each ROI time series. Hutchison, Womelsdorf, Allen, et al. (2013) noted that window sizes around 30–60 s could produce robust results of dFC; Shirer et al. reported that cognitive states may be correctly identified from the covariance matrices estimated on 30–60 s of data (Shirer, Ryali, Rykhlevskaia, Menon, & Greicius, 2012). In a series of subsequent dFC studies (Hutchison & Morton, 2015; Shen et al., 2016), the window length of 30–60 s was commonly used to achieve a tradeoff between the ability to capture FC dynamics and ensure sufficient signal‐to‐noise ratio (SNR). In our study, 176 windows were derived, within each of which an FC matrix was calculated by using Pearson's correlation of each pair of ROI signals. As the FC is reciprocal, the entries in the lower triangle (there are 160 × [160–1]/2 = 12,720 entries) of each FC matrix were extracted for each window. Finally, by concatenating all the windows, we constructed 12,720 dFC time series for each subject, each of which was 176 (number of windows) in length.

To quantify the variability of each dFC time series over time, we calculated the dFC‐ALFF for each of the 12,720 dFC time series. The dFC‐ALFF reflects the total fluctuation power of a dFC time series within a certain low‐frequency range. Of note, ALFF has been traditionally widely used to measure the fluctuations of the BOLD rs‐fMRI signals and recently, was adopted to measure the temporal variability of the dFC by our group and other researchers (Leonardi & Van De Ville, 2015; Shen et al., 2016). The dFC‐ALFF calculation includes the following steps. First, a fast Fourier transformation (FFT) of each dFC time series was conducted. Second, the ALFF was calculated by summing the amplitudes of the dFC frequency spectrum within a predefined frequency band (in this study, it is 0–1/*w* Hz, where *w* = 40 s, that is, the length of the sliding windows). Third, each dFC‐ALFF value was standardized to be a *z*‐score by subtracting the mean of all 12,720 dFC‐ALFFs of the same subject and dividing by their standard deviation. These *z*‐scores of the dFC‐ALFF were used as the features of the following associative model (Figure 1b).

### 2.4 ASB associative analysis based on whole‐brain dFC‐ALFF

We used an SVM (support vector machine)‐based regression (SVR) to associate each subject's ASB score with the dFC‐ALFF features. SVM/SVR has been widely used to handle high‐dimensional data. Soft‐margin SVM tries to find the maximal margin between two groups while allowing some misclassifications. Theoretical and experimental results have shown that such a soft margin can make the model less prone to overfitting (Ben‐Hur, Ong, Sonnenburg, Schölkopf, & Rätsch, 2008). Second, the samples are usually not linearly differentiable in the original feature space. SVM could find a decision boundary that is nonlinear in the original space but linear in a higher‐dimensional space using the “kernel trick” (Jäkel, Schölkopf, & Wichmann, 2009). SVR retains the main features of SVM with only a difference in loss penalty calculation. Here, we used an epsilon‐insensitive loss, which defines a “tube” with a width of epsilon around the regression line in the hyperspace and there is no penalty for data points far away from the regression line (Dosenbach et al., 2010).

To obtain the optimal model, we adopted a nested leave‐one‐out cross‐validation (LOOCV) strategy (Figure 1b). Because our sample size (*N* = 115) is still considered small compared to the dimensionality (12,720) of the features, LOOCV could ensure enough samples being utilized in the model training (Dosenbach et al., 2010; Finn et al., 2015). During the outer LOOCV, each sample was designated as a testing sample in turn while the remaining samples were used to train the SVR model. During the model training, we used nested inner LOOCV to obtain an optimal model. Briefly, we used inner LOOCV to optimize the *p* value from a predefined range (.001–.05, in a step of .001) and selected the model with best association performance in the inner LOOCV (the largest Pearson's correlation) as the optimal model (Figure 1b). We used a range of *p* values instead of a certain “predefined” *p* value because we were not sure about which *p* value could select the most useful feature set for ASB prediction. Although previous studies may use a single predefined *p* value, this *p* value may not be suitable in our study. On the other hand, it is the common practice for machine learning studies to use a range of parameters and optimize them, such as the *p* value here for feature selection (different *p*‐values only indicate the degree of association with ASB score), with nested LOOCV (Chen, Cai, Ryali, Supekar, & Menon, 2016; Zhou et al., 2020). In detail, in each inner LOOCV, with training samples, we calculated the correlation between dFC features and the ASB score. The features with *p* values less than the predefined *p* threshold were used to train an SVR model that was then used to predict the testing sample of the inner LOOCV. The *p* threshold that obtains the best association performance throughout the inner SVR prediction was used for predicting ASB scores with the testing data in the outer LOOCV.

In this study, the selected features from the training data were used to fit the SVR model with a linear kernel and a hyperparameter *C* = 1 (Dosenbach et al., 2010; Finn et al., 2015). We did not optimize the hyperparameter but rather fixed it because involving too many freely estimable parameters could make the model less robust (Zhou et al., 2020). For other hyperparameters, we kept them as defaults, including insensitivity = 0 and an *eInsensitive* loss function (Dosenbach et al., 2010; Finn et al., 2015). After the full outer LOOCV iterations, each of the subjects had a model‐generated ASB score. The final performance of the model was assessed by using Pearson's correlation between the model‐generated ASB scores and the real ASB scores across all the subjects. Of note, in the original CPM paper, feature selections were carried out for the positively correlated (with the behavior score) features and negatively correlated features to train different associative models, separately (Finn et al., 2015). However, we propose that both types of features should be included in the same SVR model as an “integrated model” (using both positively and negatively correlated features) to reach a relatively unbiased result.

### 2.5 Identification of the consistently associative dFC links

#### 2.5.1 Link‐wise contribution evaluation

To reveal potential imaging features that are associated with ASB, we identified the consensus dFC links that were consistently (100%) selected across all the outer LOOCV loops in the integrated model. For each consensus dFC, we further calculated the averaged Pearson's correlation between its dFC‐ALFF and the ASB score across all the outer LOOCV loops, representing the contribution of each dFC link (we hereby called it “link‐wise contribution”). Specifically, we separately evaluated the links where weakened dFC‐ALFFs were associated with severer ASB scores, and those where abnormally strengthened dFC‐ALFFs were associated with severer ASB scores.

#### 2.5.2 Region‐wise contribution evaluation

Of all the consistently selected dFC links, we then determined which brain regions were more associated with ASB. For each ROI, its “degree of contribution” was defined by a sum of half of the absolute values of the “link‐wise contributions” across all the dFC links that were connected to this region (Dosenbach et al., 2010). In this way, the ROIs with higher “degree of contributions” were deemed to be more important for ASB‐brain association.

#### 2.5.3 Network‐wise contribution evaluation

It is also important to identify which functional network(s) could be more associated with ASB. Therefore, in addition to the *link‐wise* and *region‐wise* contributions, we further summarized a “network‐level” *degree of association* for each predefined large‐scale functional network. Such a network‐level degree of association included an intra‐network, an inter‐network, and pairwise inter‐network ASB association index. The intra‐network ASB association index was calculated by a sum of the link‐wise contribution across all selected intra‐network dFC links, while an inter‐network ASB association index was calculated by the sum of the link‐wise contribution across all the dFC links connecting one network to all the other networks. If a dFC link connected two networks, half of its link‐wise contribution goes to each network. Because the ROI number in each network was different, we normalized both indices of the network‐level degree of association by multiplying its original value with *N*_max_/*N*, where *N*_max_ presents the maximum ROI number among the six networks, and N presents the ROI number of the targeting network. To investigate the contribution of special pairwise networks to the ASB‐brain association, we calculated the percentage through the ratio of the sum of the link‐wise contribution between each pairwise networks over the sum of all inter‐network link‐wise contribution.

Six well‐known functional networks were chosen from a previous study (Dosenbach et al., 2010). They are the cingulo‐opercular network (CON), frontoparietal network (FPN), default mode network (DMN), sensorimotor network (SMN), occipital network (OcN), and cerebellum network (CereN). For each network, an intra‐ and inter‐network ASB association indices were generated, and the percentage of each pairwise networks was also calculated. In this way, we generated intra‐network, inter‐network, and pairwise‐network contributions.

### 2.6 ASB association based on network‐specific dFC‐ALFF

In Section 2.4, whole‐brain dFC‐ALFFs were used to be individually associated with ASB; to further look into which brain functional network(s) might be better associated with ASB scores, we investigated *both* intra‐network connections (only the dFC‐ALFF links within the same network were used to be individually associated with ASB scores, resulting in a total of six models) *and* pairwise inter‐network connections (all the dFC‐ALFF links connecting each pair of the networks were used to be individually associated with ASB, resulting in a total of 15 models since there are 15 network pairs). For each model, we repeated the same nested‐LOOCV analysis as used in the integrated model. A threshold of *p* < 0.05 after false discovery rate (FDR) correction was adopted to indicate (a) good associative model(s).

### 2.7 Validation

To validate the effectiveness of the dFC‐based ASB association, we also used the traditional static FCs to associate with ASB scores and compared the results with the dFC‐ALFF‐based results. The difference between the two methods is that, for static FC‐based ASB association, Pearson's correlation of the entire regional rs‐fMRI signals was calculated (Shen, Wang, Liu, & Hu, 2010). For each subject, the lower triangular entries in the 160 × 160 static FC matrix were standardized to z‐scores as we did for standardizing dFC‐ALFF features. These z‐score transformed static FC features were used as features to feed into the associative model for result comparison instead of the dFC‐ALFF values.

To investigate the influence of head motion on the results, we calculated Pearson's correlation between the extent of head motion of the individuals and the 103 dFC‐ALFF features that were identified to be most contributive in the brain‐behavior association analysis. To measure head motion, we calculated the mean absolute displacement of each volume compared with the previous volume, that is, the Euclidean norm of their first‐order derivative terms ($\sqrt{\Delta x^{2}+\Delta y^{2}+\Delta z^{2}}$) (Power, Barnes, Snyder, Schlaggar, & Petersen, 2012; Zeng et al., 2014).

To test whether specific mood states can alter the brain dFC and its relationship with ASB, as suggested in Borchardt et al. (2018), Eryilmaz, Van De Ville, Schwartz, and Vuilleumier (2011), Harrison et al. (2008), Hyett et al. (2015), Kuppens, Oravecz, and Tuerlinckx (2010); Kuppens and Verduyn (2017), we used SAS scores as targets in a control correlation analysis, where SAS scores were used as targets to feed into the associative model instead of the ASB scores. The same analysis was conducted as that for dFC‐ASB association study and compared with the latter.

## 3 RESULTS

### 3.1 Performance of dFC‐ALFF‐based ASB association

**FIGURE 2:**
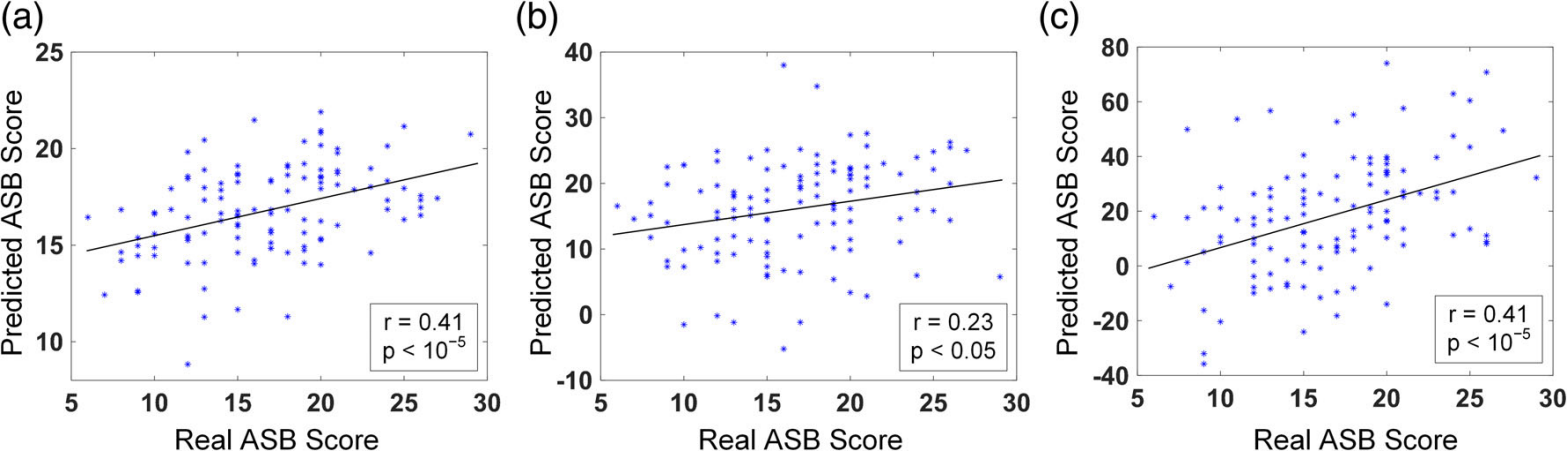
The brain‐behavior association between dFC patterns and ASB scores. (a) Scatter plot between the model‐generated and the observed ASB scores for the integrated model. (b) Scatter plot between the model‐generated and observed ASB scores for the dFC between CON and FPN. (c) Scatter plot between the model‐generated and observed ASB scores with the dFC between DMN and FPN

Based on the dFC‐ALFF, we used nested LOOCV to investigate the brain‐behavior association and found that the SVR model achieved a good performance, with *r* = .41 (*p* = 5.77 × 10^−6^) between the model‐generated and the observed ASB scores (Figure 2a).

### 3.2 Contributing dFC‐ALFF features

#### 3.2.1 Link‐wise contribution

**FIGURE 3:**
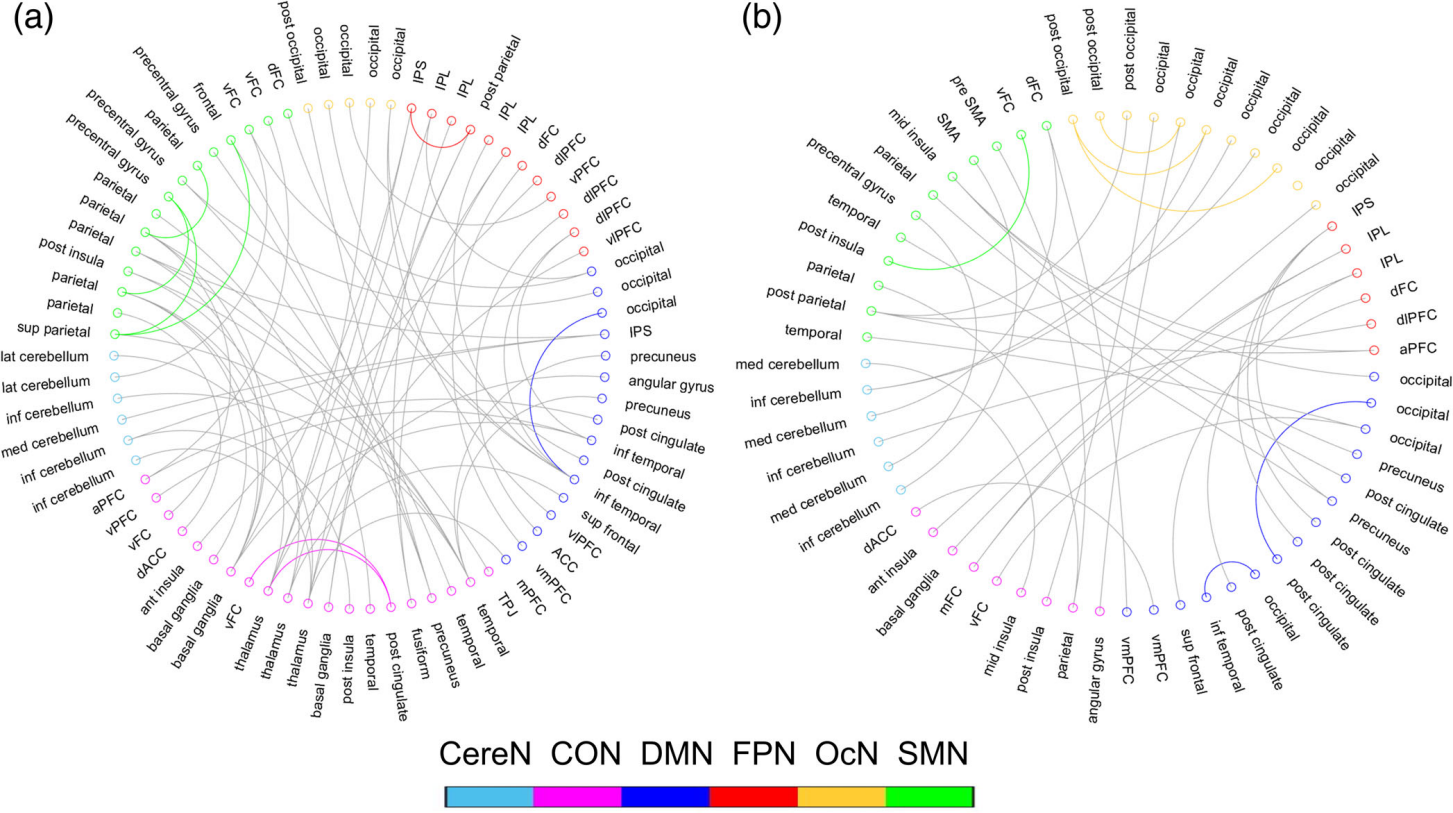
The dFC‐ALFF links that were consistently selected during ASB‐brain association, including (a) Functional connections with negative correlation with the antisocial behavior score, and (b) Functional connections with positive correlation with the antisocial behavior score. Curves in colors indicate intra‐network dFC links; gray curves indicate inter‐network dFC links. CereN, cerebellum; CON, cingulo‐opercular network; DMN, default mode network; FPN, fronto‐parietal network; OcN, occipital network; SMN, sensorimotor network

There were 66 negatively correlated features and 37 positively correlated features consistently selected across 100% outer LOOCV runs (Figure 3a,b). We found that most of the consistently contributed features were the dFC links connecting different networks (i.e., inter‐network dFC links), regardless of their positive or negative relationship with the ASB scores. Specifically, there were 54 out of 66 (81.82%) negatively correlated features and 31 out of 37 (83.78%) positively correlated features connecting different functional networks. Only a few intra‐network dFC links consistently contributed to the ASB‐brain association (Figures 3 and 5a).

#### 3.2.2 Region‐wise contribution

**FIGURE 4:**
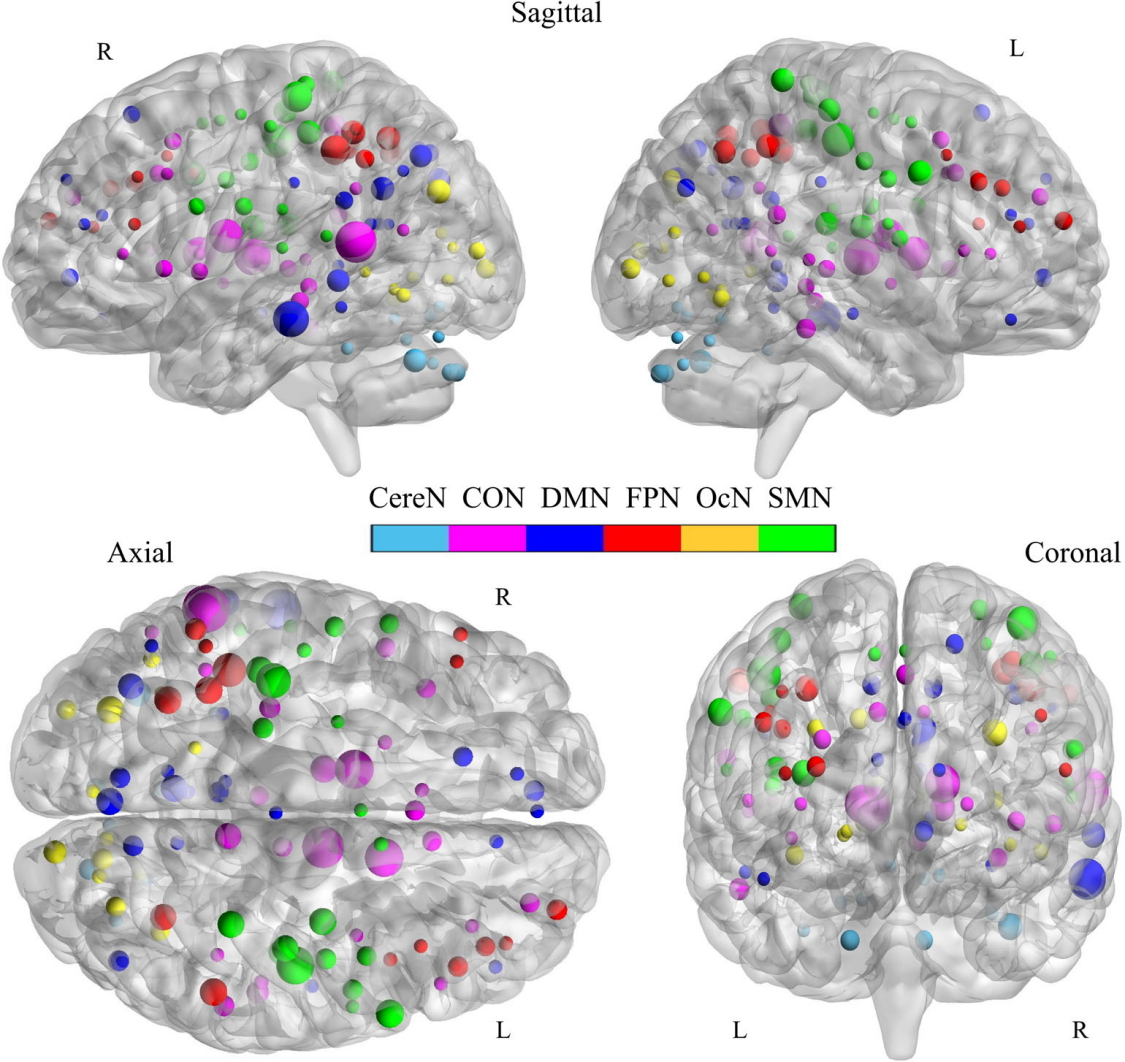
ROI‐level degree of associations with ASB scores. The size of each ROI was proportional to its ASB associative level (i.e., the degree of contributions to ASB‐brain association). For the definition of the ASB associative level, see main text. CereN, cerebellum; CON, cingulo‐opercular network; DMN, default mode network; FPN, fronto‐parietal network; L, left; OcN, occipital network; SMN, sensorimotor network; R, right

Based on the consistently selected dFC features, the ROI‐level degree of association with ASB scores was obtained for each brain region and they were visualized in Figure 4 (see Table 2 for details). In Figure 4, the size of an ROI was proportional to its ASB associative level (i.e., the degree of contribution or importance to the associative model). The most important regions were mainly located at the prefrontal cortex (PFC, including anterior, medial, dorsolateral, and ventral prefrontal areas), cingulate cortex (including anterior and posterior cingulate cortices), insula, thalamus, inferior and middle temporal cortices, and the parietal association cortex (i.e., the precuneus, inferior parietal lobule [IPL], intra‐parietal sulcus [IPS], superior parietal lobule [SPL], posterior parietal cortex [PPC]).

**TABLE 2:** Brain regions correlated with antisocial behavior scores

Brain region	MNI coordinates			Weight	Network	Brain region	MNI coordinates			Weight	Network
	*x*	*y*	*z*				*x*	*y*	*z*		
Temporal	−59	−47	11	1.73	CON	aPFC	29	57	18	0.57	FPN
Thalamus	11	−12	6	1.50	CON	dlPFC	46	28	31	0.56	FPN
Thalamus	−12	−3	13	1.36	CON	dlPFC	40	36	29	0.52	FPN
Basal ganglia	14	6	7	1.34	CON	vPFC	−52	28	17	0.30	FPN
Thalamus	−12	−12	6	0.84	CON	vlPFC	39	42	16	0.29	FPN
Precuneus	8	−40	50	0.81	CON	IPL	−48	−47	49	0.29	FPN
Fusiform	54	−31	−18	0.64	CON	dFC	44	8	34	0.28	FPN
Parietal	58	−41	20	0.59	CON	dFC	40	17	40	0.27	FPN
Post insula	−30	−28	−9	0.58	CON	dlPFC	−44	27	33	0.26	FPN
dACC	9	20	34	0.54	CON	Occipital	−29	−75	28	0.87	OcN
mFC	0	15	45	0.54	CON	Post occipital	13	−91	2	0.66	OcN
Basal ganglia	11	−24	2	0.54	CON	Occipital	15	−77	32	0.58	OcN
aPFC	27	49	26	0.52	CON	Occipital	36	−60	−8	0.54	OcN
Ant insula	−36	18	2	0.52	CON	Post occipital	−29	−88	8	0.53	OcN
vFC	−48	6	1	0.52	CON	Occipital	29	−73	29	0.52	OcN
Post cingulate	−4	−31	−4	0.52	CON	Occipital	−44	−63	−7	0.38	OcN
Angular gyrus	−41	−47	29	0.31	CON	Occipital	20	−78	−2	0.30	OcN
Temporal	51	−30	5	0.31	CON	Occipital	9	−76	14	0.30	OcN
Basal ganglia	−20	6	7	0.30	CON	Occipital	19	−66	−1	0.28	OcN
TPJ	−52	−63	15	0.28	CON	Post occipital	−5	−80	9	0.28	OcN
Basal ganglia	−6	17	34	0.28	CON	Occipital	−34	−60	−5	0.26	OcN
vFC	51	23	8	0.27	CON	Occipital	−18	−50	1	0.26	OcN
Mid insula	37	−2	−3	0.27	CON	Parietal	46	−20	45	1.32	SMN
Temporal	43	−43	8	0.26	CON	Parietal	−38	−27	60	1.19	SMN
vPFC	34	32	7	0.26	CON	dFC	60	8	34	0.89	SMN
Inf temporal	−59	−25	−15	1.46	DMN	Post parietal	−41	−31	48	0.85	SMN
Occipital	−2	−75	32	0.85	DMN	Mid insula	33	−12	16	0.84	SMN
Precuneus	−6	−56	29	0.84	DMN	Sup parietal	34	−39	65	0.84	SMN
Inf temporal	−61	−41	−2	0.80	DMN	Post insula	42	−24	17	0.82	SMN
IPS	−36	−69	40	0.79	DMN	Parietal	41	−23	55	0.82	SMN
Occipital	45	−72	29	0.58	DMN	Precentral gyrus	44	−11	38	0.58	SMN
vmPFC	−6	50	−1	0.58	DMN	Parietal	−24	−30	64	0.57	SMN
Post cingulate	−5	−43	25	0.57	DMN	Precentral gyrus	−54	−9	23	0.55	SMN
Precuneus	11	−68	42	0.56	DMN	Frontal	53	−3	32	0.55	SMN
Occipital	−9	−72	41	0.55	DMN	vFC	43	1	12	0.52	SMN
Sup frontal	−16	29	54	0.53	DMN	vFC	−55	7	23	0.51	SMN
Post cingulate	−5	−52	17	0.31	DMN	Parietal	−47	−18	50	0.31	SMN
Post cingulate	10	−55	17	0.29	DMN	Pre SMA	10	5	51	0.29	SMN
ACC	9	39	20	0.28	DMN	Temporal	−53	−37	13	0.27	SMN
vmPFC	−11	45	17	0.27	DMN	Precentral gyrus	−54	−22	22	0.26	SMN
Inf temporal	52	−15	−13	0.27	DMN	Precentral gyrus	58	−3	17	0.26	SMN
Occipital	−28	−42	−11	0.27	DMN	SMA	0	−1	52	0.26	SMN
Post cingulate	−8	−41	3	0.27	DMN	Temporal	−54	−22	9	0.25	SMN
vlPFC	46	39	−15	0.27	DMN	Parietal	−26	−8	54	0.25	SMN
mPFC	0	51	32	0.27	DMN	Inf cerebellum	−34	−67	−29	0.83	CereN
Post cingulate	1	−26	31	0.26	DMN	Inf cerebellum	18	−81	−33	0.58	CereN
Angular gyrus	−48	−63	35	0.25	DMN	Inf cerebellum	−6	−79	−33	0.53	CereN
Post cingulate	−11	−58	17	0.25	DMN	Lat cerebellum	−28	−44	−25	0.29	CereN
IPL	−41	−40	42	1.14	FPN	Med cerebellum	−11	−72	−14	0.28	CereN
IPL	54	−44	43	0.87	FPN	Inf cerebellum	33	−73	−30	0.28	CereN
Post parietal	−35	−46	48	0.86	FPN	Lat cerebellum	21	−64	−22	0.27	CereN
IPS	−32	−58	46	0.86	FPN	Med cerebellum	14	−75	−21	0.27	CereN
IPS	32	−59	41	0.82	FPN	Med cerebellum	−6	−60	−15	0.27	CereN
IPL	−53	−50	39	0.61	FPN	Med cerebellum	5	−75	−11	0.26	CereN

*Note:* Brain region names were adopted from a predefined atlas (Dosenbach et al., 2010). The related links are consistently selected by the associative model. Weight was calculated by a sum of 0.5 × absolute value of the “link‐wise contribution” across all the dFC links that connected to this ROI (Dosenbach et al., 2010). See the main text for more information.

Abbreviations: CereN, cerebellum; CON, cingulo‐opercular network; DMN, default mode network; FPN, fronto‐parietal network; OcN, occipital network; SMN, sensorimotor network.

#### 3.2.3 Network‐wise contribution

**FIGURE 5:**
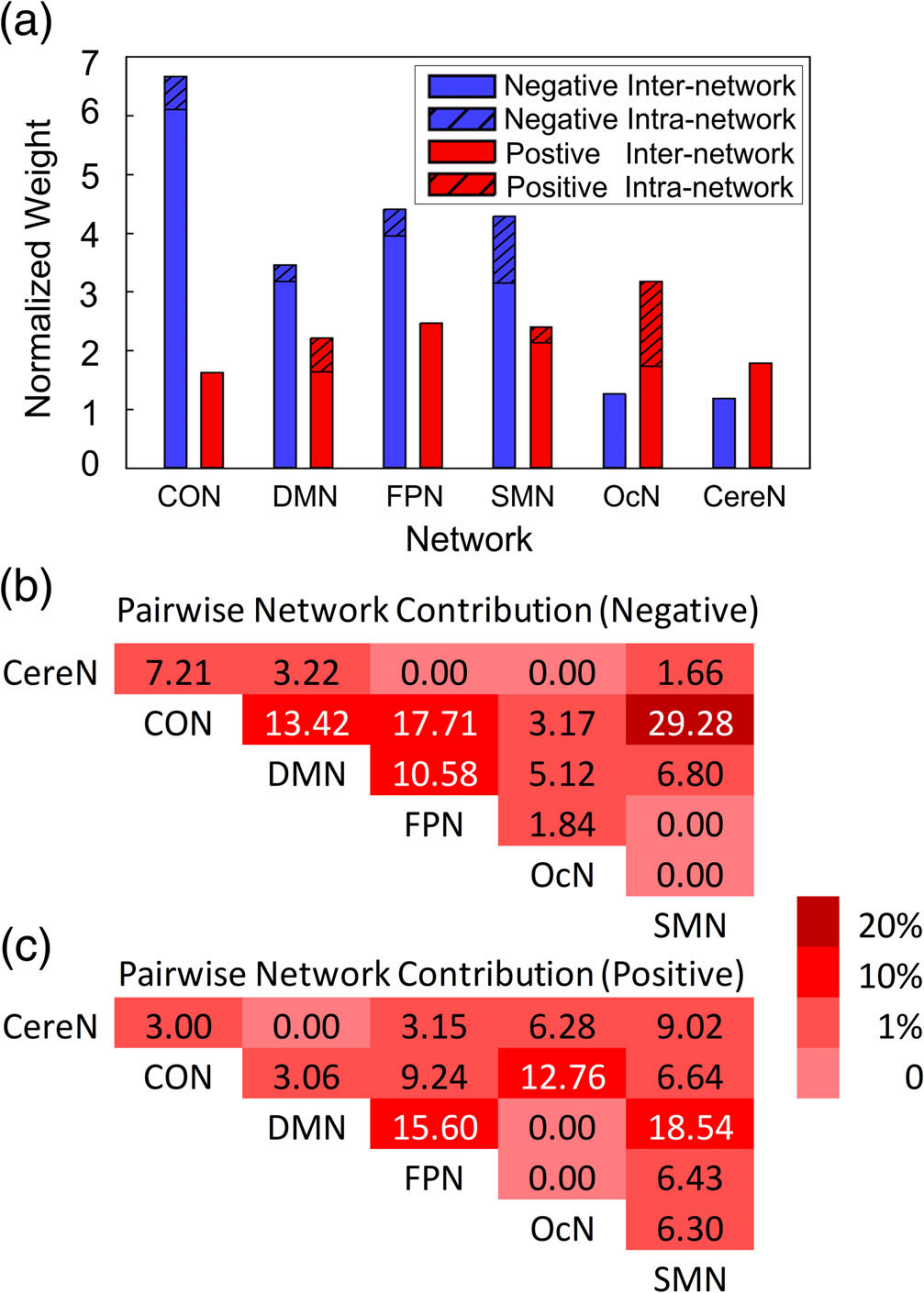
Network‐wise contribution. (a) Normalized network‐level degree of association with ASB. Blue bars indicate the network‐level contributions by the negatively correlated dFC only, and red bars indicate the network‐level contributions by the positively correlated dFC only. Dashed bars indicate contributions of intra‐network dFC, and solid bars indicate contributions of inter‐network dFC; (b) The contribution of each pairwise inter‐network connections to the ASB‐brain association by the negatively correlated dFC only; and (c) The contribution of each pairwise inter‐network connections to the ASB‐brain association by the positively correlated dFC only. CereN, cerebellum; CON, cingulo‐opercular network; DMN, default mode network; FPN, fronto‐parietal network; OcN, occipital network; SMN, sensorimotor network

The network‐level degree of association with ASB score showed more *network specificity* and more *correlation direction specificity*. That is, different large‐scale functional networks were found to differently contribute to the ASB‐brain association. Generally, the high‐order function‐related networks, such as CON (cingulo‐opercular network) and FPN (frontoparietal network), as well as one of the primary networks (sensorimotor network, or SMN), contained more negatively associated features (Figure 5a). This indicates that the severer ASB the subjects had, the lesser their dFC fluctuations were. On the other hand, the OcN (occipital network) had more positively associated features than the negatively associated ones, meaning that the greater the OcN's dFC fluctuations were, the severer ASB the subjects had. While the default mode network (DMN) and the cerebellar network (CereN) did not have such specificity regarding the positive or negative associations with the ASB scores (Figure 5a), the DMN seemed more important than the CereN in the ASB‐brain association analysis in terms of the normalized weight. Of all the six large‐scale networks, CON appeared to be more informative and more significantly associated with the ASB scores. In addition, we observed a large difference between the involvement of the inter‐network and that of the intra‐network dFC links, with the former more contributive to the ASB‐brain association than the latter.

We also calculated the percentage of the involvement to measure the possible contribution of each pairwise inter‐network connections in the ASB‐brain association (Figure 5b,c). For the negatively correlated connections, four groups of pairwise inter‐network connections were more involved in ASB‐brain association than others. If the percentage of the involvement can indicate a degree of the contribution, the inter‐network dFCs among CON, DMN, FPN, and SMN showed higher contributions than others. Among them, three pairwise inter‐network connections were related to CON (i.e., CON–DMN, CON–FPN, and CON–SMN), which revealed the CON's important role in the ASB‐brain association. The FPN and DMN may also play important roles with their high contributions identified (i.e., FPN–DMN and CON–FPN for FPN, CON–DMN, and FPN–DMN for DMN). For the positively correlated connections, DMN may play a relatively important role in the ASB‐brain association (i.e., more links of DMN–FPN and DMN–SMN were involved). In addition, CON–OcN may also present importance.

### 3.3 Performance of network‐specific ASB score association

When using intra‐network dFC links of each network to associate with ASB, none of the six networks showed any significant associative ability (*p* > .05). When using pairwise inter‐network connections to associate with ASB, the dFC between CON and FPN presented good associative ability (*r* = .23, *p* < .05, FDR corrected, Figure 2b), while the dFC between DMN and FPN showed an even better associative ability (*r* = .41, *p* < .05, FDR corrected, Figure 2c and Figure S3). Other pairwise networks did not show a significant relationship with ASB (for details, see the Supplemental Document and Table S1).

### 3.4 Performance of static FC‐based ASB association and validation

Compared to the success of the dFC‐ALFF‐based ASB association, the traditional static FC was not able to generate any valid result (*r* = −.0577, *p* = .54).

Among all the 103 contributive dFC‐ALFF features, most of them (100, 97.09%) had insignificant correlations with the extent of head motion, while only three (2.91%) showed weak correlation but they were not significant after FDR correction (Table S2). These results imply that head motion may not play an important role in our study (for details, refer the Supporting Information).

In the control correlation experiment, we used SAS scores as targets to feed into the associative model for dFC‐SAS association study. The results showed insignificant association between the dFC‐ALFF and SAS scores (*r* = −.0691, *p* = .4631; Figure S2B). From such results, we concluded that the individual emotional status as measured by SAS could not play a significant role in our study.

## 4 DISCUSSION

### 4.1 General discussions

In this study, we used temporal variations of the dFC from rs‐fMRI to investigate the ASB‐brain association. As far as we know, it is the first study that uses a dFC technique for individualized association with ASB scores. Compared to static FC, time‐varying, dynamic FC was proved to be more sensitive and informative for ASB‐brain association. A simple and intuitive dFC metric, that is, dFC‐ALFF (Leonardi & Van De Ville, 2015; Shen et al., 2016), showed its promise in individualized trait (ASB) association. More importantly, we revealed specific patterns of dFC‐ALFF associated with ASB. Based on the analysis at different spatial scales, we found that (a) ASB score was mainly negatively correlated with the degree of dFC's temporal variation, that is, severer ASB corresponds to less fluctuation of the dFC; (b) All the three high‐order cognition‐related functional networks (the DMN, CON, and FPN) might be closely associated with the behavioral abnormality in ASB subjects; (c) The inter‐network dFCs might contribute more than the intra‐network dFCs to the ASB‐brain association; and (d) One of the primary networks, SMN, seems also informative to ASB‐brain association. Our results provide useful information for the understanding of the potential neural influences on ASB, and for guiding the future interventions or treatment for subjects with severe ASB. In addition, we also found that such a brain dFC‐ASB association was less likely affected by head motion and an individual's emotional status.

### 4.2 Significantly improved ASB‐brain association based on dFC

Temporal variations of the dFC (dFC‐ALFF) were considerably better than the traditional static FC in the task of ASB‐brain association. A possible reason is that the dFC variation and the static FC amplitude may reflect different network characteristics. The dFC was proposed to be related to much higher‐level cognitive functions, such as attention levels (Madhyastha, Askren, Boord, & Grabowski, 2015), consciousness (Wang, Ong, Patanaik, Zhou, & Chee, 2016), and task adaptive control (Reddy et al., 2018). The fluctuation of the FC along time could reflect brain network flexibility (Medaglia et al., 2018). Such network flexibility could be essential for human maintenance of normative mental health (Kaiser et al., 2016; Rashid et al., 2016; Sanfratello, Houck, & Calhoun, 2019) and cognitive abilities (Chen, Cai, et al., 2016; Madhyastha et al., 2015; Xie et al., 2018). Subjects with ASB show increased impulsivity (Mackey et al., 2017), risk‐taking decisions (Syngelaki et al., 2009), and reward‐driven behavior (Murray et al., 2017); all these abnormalities could be more related to dFC. Since most of our subjects had mild ASB, their dFC could be more easily affected than the static FC.

In this study, dFC was found more sensitive to ASB scores than static FC for the mild ASB subjects. Compared to the previous studies, we found that the sensitivity could vary in different ASB‐brain association tasks. For example, our previous study has shown that static FC could be useful for classifying subjects with severe ASB (i.e., antisocial personality disorders) from normal controls (Tang et al., 2014). Such a patient versus healthy control classification task is easier than discriminating different degrees of ASB in the current regression analysis. As for disease detection sensitivity, other groups also found that dFC was more sensitive than static FC in the studies of different cognitive statuses (Kucyi & Davis, 2014; Medaglia et al., 2018; Reddy et al., 2018; Shen et al., 2016) or other brain diseases or disorders (Rashid et al., 2016; Rashid, Damaraju, Pearlson, & Calhoun, 2014; Su et al., 2016). Our study suggests that it is better to use dFC as features if the effect of interest is subtle (e.g., mental disorders at their preclinical stages).

### 4.3 Potential neural association of ASB

#### 4.3.1 Inter‐network dFC deteriorations in ASB

Regarding the brain functional connections that show dFC‐ASB correlation, one of the major findings is that the inter‐network dFC links are mainly involved. It may suggest that it is the inter‐network dFC that could be mainly associated with behavioral deficits, while the intra‐network dFCs could be largely intact and resilient (Figures 3 and 5). Such difference might indicate that ASB could mainly target inter‐network communications, therefore making the subjects manifesting difficulties in handling social behaviors. Inter‐network communications have been regarded essential in maintaining healthy executive control functions (Gallen, Turner, Adnan, & D'Esposito, 2016; Schultz & Cole, 2016). Poor executive control functions might introduce externalizing antisocial behaviors of the offenders (Klapwijk et al., 2017). On the other hand, intra‐network connections are mainly for certain specialized brain functions that are less complex than those mediated by or relying on the inter‐network connections (Gallen et al., 2016; Schultz & Cole, 2016). The largely intact intra‐network dFCs could be the reason that these ASB subjects still look normal in a general sense.

The network‐specific ASB association revealed three higher‐order networks (CON, FPN, and DMN) that were more strongly associated with ASB than the other functional networks. Interestingly, these three networks have also been mentioned and studied as a triple network model (Menon, 2011), which relies on the assumption that the aberrant inter‐network connections could underlie a wide range of psychiatric and neurological disorders (Wu et al., 2016). Our results showed that the temporal variability of the inter‐network dFCs, especially those among the three networks, might be more important in maintaining normative cognitive functions and affective statuses, such as healthy social behaviors. Therefore, we provided another support to the importance of the triple network model from the dFC point of view.

#### 4.3.2 Disruptions of the network‐specific dFC

The CON and FPN are considered as two major networks related to control functions (Dosenbach et al., 2010). In our study, both CON and FPN showed less dFC variability with each other and with other networks for the subjects with severer ASB. These two control networks may contribute to the flexible control of the goal‐directed behaviors, such as executive control, attentional control, and emotional control through their moment‐to‐moment interactions and those with other regions (Coste & Kleinschmidt, 2016; Dosenbach et al., 2010; Dosenbach, Fair, Cohen, Schlaggar, & Petersen, 2008; Sadaghiani & D'Esposito, 2015). The CON was believed to provide stable task control over entire task epochs, while the FPN seems to be able to initiate and adjust control in response to feedbacks (Dosenbach et al., 2008; Duncan, 2010; Spreng, Sepulcre, Turner, Stevens, & Schacter, 2013). In a recent study investigating executive functions, the CON and FPN were consistently engaged across switching, updating, and inhibiting tasks (Engelhardt, Harden, Tucker‐Drob, & Church, 2018). The impaired dFC variability between the two control networks and between them and other networks in the ASB subjects may result in insufficient control and dysregulation of behaviors, thus leading to more impulsive or involuntary behaviors.

We found both positively and negatively correlated DMN‐related dFC with ASB scores, indicating its complex relationship to the abnormal behaviors. The DMN's functionality and its interaction with the control systems have been proposed to play an important role in regulating a wide range of internal thoughts (Andrews‐Hanna, Smallwood, & Spreng, 2014; Buckner & DiNicola, 2019; Spreng, Mar, & Kim, 2009), emotion regulation, future planning, and self‐inspection (Mak et al., 2017). Apathy, defined as loss or reduction of motivation and associated with decreases in at least two out of three of goal‐directed behavior, cognitive activity, or emotion (Husain & Roiser, 2018), has been regarded mainly related to the alterations of the DMN (Husain & Roiser, 2018). However, the statistical DMN fluctuations reported in those studies were the product of contrasting between two or more conditions (i.e., a target condition vs. a baseline condition, or a healthy status vs. a diseased status). Here, we revealed the association between DMN's dFC fluctuations and continuous ASB scores. Specifically, we found that the DMN's FC trajectories within a few minutes or the DMN‐related FC's temporal dynamics were associated with ASB scores, which may be correlated with altered high‐order functions carried out by DMN, such as apathy, self‐inspection deficiency, absence of long‐term planning, and difficulty with behavior control in ASB individuals.

The SMN is the only primary functional system that was found to be responsible for ASB, where its dFC variability with CON was found to be negatively associated with ASB, while its dFC variability with DMN was found to be positively associated with ASB. This might imply that it is difficult for the ASB subjects to receive control information sent from these high‐level networks to maintain behavioral adaption to the fast‐changing environment. It is also possibly associated with abnormal behavioral responses, which could be externalized as antisocial behavior performance, including impulsivity, risk‐taking, violent, aggressive behavior, and affective instability.

It should be noted that, due to the nature of multivariate machine learning analysis with nested LOOCV, the predictive results and the contributive features between the whole brain‐based and specific network‐based ASB associations were not strictly the same. We used embedded‐ (or nested‐) LOOCV to select features and the selected features were fed into an SVR model for training the association model. When different features were considered, the complex relationship among them also changed, which resulted in different classification hyperplane and “weights” on the selected features derived from the SVR training. This multivariate analysis will generate different results compared to the mass‐univariate analysis such as regression (as each link will be evaluated independently in the latter method). Nevertheless, we found that the results are still consistent to a large extent. On one hand, almost all the predictive links are inter‐network links based on whole‐brain‐based association analysis, which is consistent with the network‐wise association analysis with only significant inter‐network link‐based associations. On the other hand, the CON, DMN, and FPN are among the most useful functional networks compared to the others (Figure 5a), which is consistent with the network‐wise association results (only links between CON and FPN, as well as those between DMN and FPN, show significant associative abilities with the ASB scores; Figure 2b,c).

### 4.4 Possible compensatory effect of cognitive function damage in ASB

In our study, we found that several connectivities showed positively correlated dFC variation with ASB severity, which might indicate a possible compensatory effect against the impaired executive control functions in ASB subjects. These dynamic links are mainly inter‐network connections (Figures 3 and 5). Neuroscience research has revealed strong brain plasticity in motor, sensory, and cognitive domains, even in the social capacities (Valk et al., 2017). This type of functional compensation is considered as a neural plastic effect of structural or functional disturbances (Iraji et al., 2016; Tomassini et al., 2016). Therefore, we assume that these increased dFC links in the subjects with severer behavioral disturbance are more likely to be related to functional compensation.

### 4.5 Limitations and future work

In this study, the low‐frequency fluctuations of dFC were used as features to be associated with ASB scores. This metric characterizes the temporal variability of the dFC but might ignore other rich information in the dFC, such as the covariance among different dFC time series (i.e., “high‐order FC”; Chen et al., 2016). The sample size of the mild ASB participants is also relatively small for the associative analysis. We will carry out a further experiment and use a large sample size to validate our findings. Additionally, we followed the previous studies to perform within‐subject standardization to the dFC‐ALFF features, because it is suggested for reducing undesirable relationships with nuisance variables and increasing test–retest reliability of ALFF metrics (Yan, Craddock, Zuo, Zang, & Milham, 2013). In the future, we will investigate the differences between subject‐specific z‐scoring and other commonly used feature standardization methods in the machine learning field. Since resting state is a less constrained “status” where it is difficult to monitor or maintain subjects' status during the rs‐fMRI scan, the interpretation of the rs‐fMRI‐based findings should be carefully made. Many studies have indicated that varying emotional or cognitive events during the so‐called “resting state” could modulate dFC (Barnes, Bullmore, & Suckling, 2009; Cole, Bassett, Power, Braver, & Petersen, 2014; Gonzalez‐Castillo et al., 2015; Grigg & Grady, 2010). However, due to technical limitations, monitoring subjects' emotional and cognitive statuses during rs‐fMRI scan was not achieved at the time when the data was collected. More careful experimental design, such as monitoring and controlling emotional and cognitive statuses, should be conducted in our future study. Finally, we did not investigate whether the associations between brain dFC and ASB may be modulated by the severity of ASB. Such a study, however, is interesting and will be investigated in the future when we enrolled enough samples.

## 5 CONCLUSIONS

Our study demonstrated the effectiveness of using dynamic FC in antisocial behavior severity association in a machine learning framework. Dynamic neural circuit disruptions were found to possibly be associated with less cognitive control and more antisocial behavior, such as the inter‐network dynamic FCs between the high‐level control networks and other functional systems. In particular, three high‐order cognitive function‐related networks showed to be associated with ASB, especially the impaired controllability as reflected by the reduced dFCs among the control networks. Our study demonstrates that the temporal FC variation measured by the dFC‐ALFF method could be a promising tool for ASB assessment, treatment, and prevention.

## CONFLICT OF INTEREST

The authors declare no conflict of interest. Dinggang Shen was involved in this work but he is now in a full‐time position with a new affiliation (United Imaging Intelligence, a subsidiary of United Imaging Healthcare Co., Ltd.).

## Supporting information


**FIGURE S1** The distribution of APSD scores. APSD: Antisocial Process Screening Device
**FIGURE S2**. (a) The distribution of SAS scores; (b) Scatter plot between the model‐generated and the observed SAS scores for the integrated model. SAS: Self‐rating Anxiety Scale
**TABLE S1**. Network‐wise ASB Score Association
**FIGURE S3**. Scatter plot between the model‐generated and observed ASB scores with the dFC between DMN and FPN.
**TABLE S2**. The Pearson correlation between the 103 contributive dFC‐ALFF features and the extent of head motion

## Data Availability

Due to sensitive patient information, the fMRI data is protected by the Third Xiangya Hospital of Central South University and thus cannot be made publicly available. However, upon direct request, we will be able to share all our code for connectome‐based predictive modeling for boosting future research.

## References

[hbm25225-bib-0001] Allen, E. A. , Damaraju, E. , Plis, S. M. , Erhardt, E. B. , Eichele, T. , & Calhoun, V. D. (2014). Tracking whole‐brain connectivity dynamics in the resting state. Cerebral Cortex, 24(3), 663–676. 10.1093/cercor/bhs352 23146964 PMC3920766

[hbm25225-bib-0002] Andrews‐Hanna, J. R. , Smallwood, J. , & Spreng, R. N. (2014). The default network and self‐generated thought: Component processes, dynamic control, and clinical relevance. Annals of the New York Academy of Sciences, 1316(1), 29–52. 10.1111/nyas.12360 24502540 PMC4039623

[hbm25225-bib-0003] Barnes, A. , Bullmore, E. T. , & Suckling, J. (2009). Endogenous human brain dynamics recover slowly following cognitive effort. PLoS One, 4(8), e6626 10.1371/journal.pone.0006626 19680553 PMC2721686

[hbm25225-bib-0004] Ben‐Hur, A. , Ong, C. S. , Sonnenburg, S. , Schölkopf, B. , & Rätsch, G. (2008). Support vector machines and kernels for computational biology. PLoS Computational Biology, 4(10), e1000173 10.1371/journal.pcbi.1000173 18974822 PMC2547983

[hbm25225-bib-0005] Betti, V. , Della Penna, S. , de Pasquale, F. , Mantini, D. , Marzetti, L. , Romani, G. L. , & Corbetta, M. (2013). Natural scenes viewing alters the dynamics of functional connectivity in the human brain. Neuron, 79(4), 782–797. 10.1016/j.neuron.2013.06.022 23891400 PMC3893318

[hbm25225-bib-0006] Blair, R. J. (2012). Cortical thinning and functional connectivity in psychopathy. The American Journal of Psychiatry, 169(7), 684–687. 10.1176/appi.ajp.2012.12030396 22760187

[hbm25225-bib-0007] Borchardt, V. , Fan, Y. , Dietz, M. , Melendez, A. L. H. , Bajbouj, M. , Gärtner, M. , … Grimm, S. (2018). Echoes of affective stimulation in brain connectivity networks. Cerebral Cortex, 28(12), 4365–4378. 10.1093/cercor/bhx290 29161351

[hbm25225-bib-0008] Buckner, R. L. , & DiNicola, L. M. (2019). The brain's default network: Updated anatomy, physiology and evolving insights. Nature Reviews. Neuroscience, 20(10), 593–608. 10.1038/s41583-019-0212-7 31492945

[hbm25225-bib-0009] Chen, T. , Cai, W. , Ryali, S. , Supekar, K. , & Menon, V. (2016). Distinct global brain dynamics and spatiotemporal Organization of the Salience Network. PLoS Biology, 14(6), e1002469 10.1371/journal.pbio.1002469 27270215 PMC4896426

[hbm25225-bib-0010] Chen, X. , Zhang, H. , Gao, Y. , Wee, C. Y. , Li, G. , & Shen, D. (2016). High‐order resting‐state functional connectivity network for MCI classification. Human Brain Mapping, 37(9), 3282–3296. 10.1002/hbm.23240 27144538 PMC4980261

[hbm25225-bib-0011] Chen, X. , Zhang, H. , Zhang, L. , Shen, C. , Lee, S. W. , & Shen, D. (2017). Extraction of dynamic functional connectivity from brain grey matter and white matter for MCI classification. Human Brain Mapping, 38(10), 5019–5034. 10.1002/hbm.23711 28665045 PMC5593789

[hbm25225-bib-0012] Cole, M. W. , Bassett, D. S. , Power, J. D. , Braver, T. S. , & Petersen, S. E. (2014). Intrinsic and task‐evoked network architectures of the human brain. Neuron, 83(1), 238–251. 10.1016/j.neuron.2014.05.014 24991964 PMC4082806

[hbm25225-bib-0013] Contreras‐Rodríguez, O. , Pujol, J. , Batalla, I. , Harrison, B. J. , Soriano‐Mas, C. , Deus, J. , … Hernández‐Ribas, R. (2015). Functional connectivity bias in the prefrontal cortex of psychopaths. Biological Psychiatry, 78(9), 647–655. 10.1016/j.biopsych.2014.03.007 24742618

[hbm25225-bib-0014] Coste, C. P. , & Kleinschmidt, A. (2016). Cingulo‐opercular network activity maintains alertness. NeuroImage, 128, 264–272. 10.1016/j.neuroimage.2016.01.026 26801604

[hbm25225-bib-0015] de Wied, M. , van der Baan, H. , Raaijmakers, Q. , de Ruiter, C. , & Meeus, W. (2014). Factor structure and construct validity of the Dutch version of the antisocial process screening device. Journal of Psychopathology and Behavioral Assessment, 36(1), 84–92. 10.1007/s10862-013-9371-4

[hbm25225-bib-0016] Dosenbach, N. U. , Fair, D. A. , Cohen, A. L. , Schlaggar, B. L. , & Petersen, S. E. (2008). A dual‐networks architecture of top‐down control. Trends in Cognitive Sciences, 12(3), 99–105. 10.1016/j.tics.2008.01.001 18262825 PMC3632449

[hbm25225-bib-0017] Dosenbach, N. U. , Nardos, B. , Cohen, A. L. , Fair, D. A. , Power, J. D. , Church, J. A. , … Schlaggar, B. L. (2010). Prediction of individual brain maturity using fMRI. Science, 329(5997), 1358–1361. 10.1126/science.1194144 20829489 PMC3135376

[hbm25225-bib-0018] Duncan, J. (2010). The multiple‐demand (MD) system of the primate brain: Mental programs for intelligent behaviour. Trends in Cognitive Sciences, 14(4), 172–179. 10.1016/j.tics.2010.01.004 20171926

[hbm25225-bib-0019] Dunstan, D. A. , & Scott, N. (2018). Assigning clinical significance and symptom severity using the Zung scales: Levels of misclassification arising from confusion between index and raw scores. Depression Research and Treatment, 2018, 1–13. 10.1155/2018/9250972 PMC5828114 29610683

[hbm25225-bib-0020] Engelhardt, L. E. , Harden, K. P. , Tucker‐Drob, E. M. , & Church, J. A. (2018). The neural architecture of executive functions is established by middle childhood. Neuroimage, 185, 479–489. 10.1016/j.neuroimage.2018.10.024 30312810 PMC6366457

[hbm25225-bib-0021] Eryilmaz, H. , Van De Ville, D. , Schwartz, S. , & Vuilleumier, P. (2011). Impact of transient emotions on functional connectivity during subsequent resting state: A wavelet correlation approach. NeuroImage, 54(3), 2481–2491. 10.1016/j.neuroimage.2010.10.021 20955802

[hbm25225-bib-0111] Fan, Y. , Rao, H. , Hurt, H. , Giannetta, J. , Korczykowski, M. , Shera, D. , … Dinggang, S. (2007). Multivariate examination of brain abnormality using both structural and functional MRI. Neuroimage, 36(4), 1189–1199. 10.1016/j.neuroimage.2007.04.009 17512218

[hbm25225-bib-0110] Fan, Y. , Gur, E. R. , Gur, C. R. , Wu, X. , Shen, D. , Calkins, E. M. , & Davatzikos, C. (2008). Unaffected family members and schizophrenia patients share brain structure patterns: a high‐dimensional pattern classification study. Biol Psychiatry, 63(1), 118–124. 10.1016/j.biopsych.2007.03.015 17555720 PMC2190626

[hbm25225-bib-0022] Finn, E. S. , Shen, X. L. , Scheinost, D. , Rosenberg, M. D. , Huang, J. , Chun, M. M. , … Constable, R. T. (2015). Functional connectome fingerprinting: Identifying individuals using patterns of brain connectivity. Nature Neuroscience, 18(11), 1664–1671. 10.1038/nn.4135 26457551 PMC5008686

[hbm25225-bib-0023] Fong, A. H. C. , Yoo, K. , Rosenberg, M. D. , Zhang, S. , Li, C. R. , Scheinost, D. , … Chun, M. M. (2019). Dynamic functional connectivity during task performance and rest predicts individual differences in attention across studies. NeuroImage, 188, 14–25. 10.1016/j.neuroimage.2018.11.057 30521950 PMC6401236

[hbm25225-bib-0024] Frick, P. J. , & Hare, R. D. (2001). Antisocial process screening device: APSD. Multi‐Health Systems Toronto. Retrieved from http://www.hare.org/scales/apsd.html.

[hbm25225-bib-0025] Gallen, C. L. , Turner, G. R. , Adnan, A. , & D'Esposito, M. (2016). Reconfiguration of brain network architecture to support executive control in aging. Neurobiology of Aging, 44, 42–52. 10.1016/j.neurobiolaging.2016.04.003 27318132 PMC4913038

[hbm25225-bib-0026] Gonzalez‐Castillo, J. , Hoy, C. W. , Handwerker, D. A. , Robinson, M. E. , Buchanan, L. C. , Saad, Z. S. , & Bandettini, P. A. (2015). Tracking ongoing cognition in individuals using brief, whole‐brain functional connectivity patterns. Proceedings of the National Academy of Sciences of the United States of America, 112(28), 8762–8767. 10.1073/pnas.1501242112 26124112 PMC4507216

[hbm25225-bib-0027] Goodwin, B. E. , Sellbom, M. , & Salekin, R. T. (2015). Elucidating the construct validity of the antisocial process screening device (APSD) in a sample of young adults. Journal of Psychopathology and Behavioral Assessment, 37(1), 1–11. 10.1007/s10862-014-9444-z

[hbm25225-bib-0028] Goulter, N. , Kimonis, E. R. , & Heller, E. (2018). Antisocial process screening device subscales predict recidivism in an Australian juvenile offender sample. Journal of Psychopathology and Behavioral Assessment, 40(2), 159–168. 10.1007/s10862-018-9669-3

[hbm25225-bib-0029] Grigg, O. , & Grady, C. L. (2010). The default network and processing of personally relevant information: Converging evidence from task‐related modulations and functional connectivity. Neuropsychologia, 48(13), 3815–3823. 10.1016/j.neuropsychologia.2010.09.007 20837034 PMC3104039

[hbm25225-bib-0030] Harenski, C. L. , Harenski, K. A. , Shane, M. S. , & Kiehl, K. A. (2010). Aberrant neural processing of moral violations in criminal psychopaths. Journal of Abnormal Psychology, 119(4), 863–874. 10.1037/a0020979 21090881 PMC3985413

[hbm25225-bib-0031] Harrison, B. J. , Pujol, J. , Ortiz, H. , Fornito, A. , Pantelis, C. , & Yücel, M. (2008). Modulation of brain resting‐state networks by sad mood induction. PLoS One, 3(3), e1794 10.1371/journal.pone.0001794 18350136 PMC2263138

[hbm25225-bib-0032] Husain, M. , & Roiser, J. P. (2018). Neuroscience of apathy and anhedonia: A transdiagnostic approach. Nature Reviews. Neuroscience, 19(8), 470–484. 10.1038/s41583-018-0029-9 29946157

[hbm25225-bib-0033] Hutchison, R. M. , & Morton, J. B. (2015). Tracking the Brain's functional coupling dynamics over development. The Journal of Neuroscience, 35(17), 6849–6859. 10.1523/JNEUROSCI.4638-14.2015 25926460 PMC6605187

[hbm25225-bib-0034] Hutchison, R. M. , & Morton, J. B. (2016). It's a matter of time: Reframing the development of cognitive control as a modification of the brain's temporal dynamics. Developmental Cognitive Neuroscience, 18, 70–77. 10.1016/j.dcn.2015.08.006 26375924 PMC6990064

[hbm25225-bib-0035] Hutchison, R. M. , Womelsdorf, T. , Allen, E. A. , Bandettini, P. A. , Calhoun, V. D. , Corbetta, M. , … Chang, C. (2013). Dynamic functional connectivity: Promise, issues, and interpretations. NeuroImage, 80, 360–378. 10.1016/j.neuroimage.2013.05.079 23707587 PMC3807588

[hbm25225-bib-0036] Hutchison, R. M. , Womelsdorf, T. , Gati, J. S. , Everling, S. , & Menon, R. S. (2013). Resting‐state networks show dynamic functional connectivity in awake humans and anesthetized macaques. Human Brain Mapping, 34(9), 2154–2177. 10.1002/hbm.22058 22438275 PMC6870538

[hbm25225-bib-0037] Hyett, M. P. , Parker, G. B. , Guo, C. C. , Zalesky, A. , Nguyen, V. T. , Yuen, T. , & Breakspear, M. (2015). Scene unseen: Disrupted neuronal adaptation in melancholia during emotional film viewing. NeuroImage: Clinical, 9, 660–667. 10.1016/j.nicl.2015.10.011 26740919 PMC4660155

[hbm25225-bib-0038] Iraji, A. , Chen, H. B. , Wiseman, N. , Welch, R. D. , O'Neil, B. J. , Haacke, E. M. , … Kou, Z. F. (2016). Compensation through functional hyperconnectivity: A longitudinal connectome assessment of mild traumatic brain injury. Neural Plasticity, 2016, 4072402 10.1155/2016/4072402 26819765 PMC4706919

[hbm25225-bib-0039] Jäkel, F. , Schölkopf, B. , & Wichmann, F. A. (2009). Does cognitive science need kernels? Trends in Cognitive Sciences, 13(9), 381–388. 10.1016/j.tics.2009.06.002 19729333

[hbm25225-bib-0040] Jiang, W. , Li, G. , Liu, H. , Shi, F. , Wang, T. , Shen, C. , … Wang, W. (2016). Reduced cortical thickness and increased surface area in antisocial personality disorder. Neuroscience, 337, 143–152. 10.1016/j.neuroscience.2016.08.052 27600947 PMC5152675

[hbm25225-bib-0041] Jiang, W. , Shi, F. , Liu, H. , Li, G. , Ding, Z. , Shen, H. , … Wang, W. (2017). Reduced white matter integrity in antisocial personality disorder: A diffusion tensor imaging study. Scientific Reports, 7, 43002 10.1038/srep43002 28223713 PMC5320449

[hbm25225-bib-0042] Jie, B. , Liu, M. , & Shen, D. (2018). Integration of temporal and spatial properties of dynamic connectivity networks for automatic diagnosis of brain disease. Medical Image Analysis, 47, 81–94. 10.1016/j.media.2018.03.013 29702414 PMC5986611

[hbm25225-bib-0043] Kaiser, R. H. , Whitfield‐Gabrieli, S. , Dillon, D. G. , Goer, F. , Beltzer, M. , Minkel, J. , … Pizzagalli, D. A. (2016). Dynamic resting‐state functional connectivity in major depression. Neuropsychopharmacology, 41(7), 1822–1830. 10.1038/npp.2015.352 26632990 PMC4869051

[hbm25225-bib-0044] Klapwijk, E. T. , van den Bos, W. , & Güroğlu, B. (2017). Neural mechanisms of criminal decision making in adolescence. The Oxford Handbook of Offender Decision Making, 6, 246.

[hbm25225-bib-0045] Kucyi, A. , & Davis, K. D. (2014). Dynamic functional connectivity of the default mode network tracks daydreaming. NeuroImage, 100, 471–480. 10.1016/j.neuroimage.2014.06.044 24973603

[hbm25225-bib-0046] Kucyi, A. , Salomons, T. V. , & Davis, K. D. (2013). Mind wandering away from pain dynamically engages antinociceptive and default mode brain networks. Proceedings of the National Academy of Sciences of the United States of America, 110(46), 18692–18697. 10.1073/pnas.1312902110 24167282 PMC3832014

[hbm25225-bib-0047] Kuppens, P. , Oravecz, Z. , & Tuerlinckx, F. (2010). Feelings change: Accounting for individual differences in the temporal dynamics of affect. Journal of Personality and Social Psychology, 99(6), 1042 10.1037/a0020962 20853980

[hbm25225-bib-0048] Kuppens, P. , & Verduyn, P. (2017). Emotion dynamics. Current Opinion in Psychology, 17, 22–26. 10.1016/j.copsyc.2017.06.004 28950968

[hbm25225-bib-0049] Leonardi, N. , & Van De Ville, D. (2015). On spurious and real fluctuations of dynamic functional connectivity during rest. NeuroImage, 104, 430–436. 10.1016/j.neuroimage.2014.09.007 25234118

[hbm25225-bib-0050] Li, X. , Chan, W. T. , Ang, R. P. , & Huan, V. S. (2017). Assessment of psychopathic traits in Singaporean adolescents: Validation of the antisocial process screening device (APSD). Journal of Psychopathology and Behavioral Assessment, 39(2), 198–208. 10.1007/s10862-016-9579-1

[hbm25225-bib-0051] Li, X. , Zang, Y.‐F. , & Zhang, H. (2015). Exploring dynamic brain functional networks using continuous “state‐related” functional MRI. BioMed Research International, 2015, 824710 10.1155/2015/824710 26413546 PMC4564637

[hbm25225-bib-0052] Liao, W. , Zhang, Z. Q. , Mantini, D. , Xu, Q. , Ji, G. J. , Zhang, H. , … Lu, G. M. (2014). Dynamical intrinsic functional architecture of the brain during absence seizures. Brain Structure & Function, 219(6), 2001–2015. 10.1007/s00429-013-0619-2 23913255

[hbm25225-bib-0053] Liu, L. , Zhang, H. , Wu, J. , Yu, Z. , Chen, X. , Rekik, I. , … Shen, D. (2018). Overall survival time prediction for high‐grade glioma patients based on large‐scale brain functional networks. Brain Imaging and Behavior, 13(5), 1–19. 10.1007/s11682-018-9949-2 30155788

[hbm25225-bib-0054] Lurie, D. , Kessler, D. , Bassett, D. , Betzel, R. F. , Breakspear, M. , Keilholz, S. , … McIntosh, A. R. (2018). On the nature of resting fMRI and time‐varying functional connectivity. 10.31234/osf.io/xtzre PMC7006871 32043043

[hbm25225-bib-0112] Luan, H. , Qi, F. , Xue, Z. , Chen, L. , & Shen, D. (2008). Multimodality image registration by maximization of quantitative–qualitative measure of mutual information. Pattern Recognition, 41(1), 285–298. 10.1016/j.patcog.2007.04.002

[hbm25225-bib-0055] Ly, M. , Motzkin, J. C. , Philippi, C. L. , Kirk, G. R. , Newman, J. P. , Kiehl, K. A. , & Koenigs, M. (2012). Cortical thinning in psychopathy. The American Journal of Psychiatry, 169(7), 743–749. 10.1176/appi.ajp.2012.11111627 22581200 PMC3815681

[hbm25225-bib-0056] Mackey, S. , Chaarani, B. , Kan, K. J. , Spechler, P. A. , Orr, C. , Banaschewski, T. , … Garavan, H. (2017). Brain regions related to impulsivity mediate the effects of early adversity on antisocial behavior. Biological Psychiatry, 82(4), 275–282. 10.1016/j.biopsych.2015.12.027 26971049

[hbm25225-bib-0057] Madhyastha, T. M. , Askren, M. K. , Boord, P. , & Grabowski, T. J. (2015). Dynamic connectivity at rest predicts attention task performance. Brain Connectivity, 5(1), 45–59. 10.1089/brain.2014.0248 25014419 PMC4313397

[hbm25225-bib-0058] Mak, L. E. , Minuzzi, L. , MacQueen, G. , Hall, G. , Kennedy, S. H. , & Milev, R. (2017). The default mode network in healthy individuals: A systematic review and meta‐analysis. Brain Connectivity, 7(1), 25–33. 10.1089/brain.2016.0438 27917679

[hbm25225-bib-0059] Marsh, A. A. , & Cardinale, E. M. (2014). When psychopathy impairs moral judgments: Neural responses during judgments about causing fear. Social Cognitive and Affective Neuroscience, 9(1), 3–11. 10.1093/scan/nss097 22956667 PMC3871724

[hbm25225-bib-0060] Medaglia, J. D. , Satterthwaite, T. D. , Kelkar, A. , Ciric, R. , Moore, T. M. , Ruparel, K. , … Bassett, D. S. (2018). Brain state expression and transitions are related to complex executive cognition in normative neurodevelopment. NeuroImage, 166, 293–306. 10.1016/j.neuroimage.2017.10.048 29126965 PMC5747984

[hbm25225-bib-0061] Menon, V. (2011). Large‐scale brain networks and psychopathology: A unifying triple network model. Trends in Cognitive Sciences, 15(10), 483–506. 10.1016/j.tics.2011.08.003 21908230

[hbm25225-bib-0062] Murray, L. , Shaw, D. S. , Forbes, E. E. , & Hyde, L. W. (2017). Reward‐related neural correlates of antisocial behavior and callous‐unemotional traits in young men. Biological Psychiatry: Cognitive Neuroscience and Neuroimaging, 2(4), 346–354. 10.1016/j.bpsc.2017.01.009 28944306 PMC5606223

[hbm25225-bib-0063] Murray, L. , Waller, R. , & Hyde, L. (2018). A systematic review examining the link between psychopathic personality traits, antisocial behavior, and neural reactivity during reward and loss processing. Personality Disorders, 9(6), 497 10.1037/per0000308 30080060 PMC7238432

[hbm25225-bib-0064] Narayan, V. M. , Narr, K. L. , Kumari, V. , Woods, R. P. , Thompson, P. M. , Toga, A. W. , & Sharma, T. (2007). Regional cortical thinning in subjects with violent antisocial personality disorder or schizophrenia. The American Journal of Psychiatry, 164(9), 1418–1427. 10.1176/appi.apj.2007.06101631 17728428 PMC3197838

[hbm25225-bib-0065] Ogilvie, J. M. , Stewart, A. L. , Chan, R. C. K. , & Shum, D. H. K. (2011). Neuropsychological measures of executive function and antisocial behavior: A meta‐analysis*. Criminology, 49(4), 1063–1107. 10.1111/j.1745-9125.2011.00252.x

[hbm25225-bib-0066] Pechorro, P. , Maroco, J. , Poiares, C. , & Vieira, R. X. (2013). Validation of the Portuguese version of the antisocial process screening device‐self‐report with a focus on delinquent behavior and behavior problems. International Journal of Offender Therapy and Comparative Criminology, 57(1), 112–126. 10.1177/0306624X11427174 22094596

[hbm25225-bib-0067] Poldrack, R. A. , Monahan, J. , Imrey, P. B. , Reyna, V. , Raichle, M. E. , Faigman, D. , & Buckholtz, J. W. (2017). Predicting violent behavior: What can neuroscience add? Trends in Cognitive Sciences, 22(2), 111–123. 10.1016/j.tics.2017.11.003 29183655 PMC5794654

[hbm25225-bib-0068] Power, J. D. , Barnes, K. A. , Snyder, A. Z. , Schlaggar, B. L. , & Petersen, S. E. (2012). Spurious but systematic correlations in functional connectivity MRI networks arise from subject motion. NeuroImage, 59(3), 2142–2154. 10.1016/j.neuroimage.2011.10.018 22019881 PMC3254728

[hbm25225-bib-0069] Preti, M. G. , Bolton, T. A. , & Van De Ville, D. (2017). The dynamic functional connectome: State‐of‐the‐art and perspectives. NeuroImage, 160, 41–54. 10.1016/j.neuroimage.2016.12.061 28034766

[hbm25225-bib-0070] Pujol, J. , Batalla, I. , Contreras‐Rodriguez, O. , Harrison, B. J. , Pera, V. , Hernandez‐Ribas, R. , … Cardoner, N. (2012). Breakdown in the brain network subserving moral judgment in criminal psychopathy. Social Cognitive and Affective Neuroscience, 7(8), 917–923. 10.1093/scan/nsr075 22037688 PMC3501707

[hbm25225-bib-0071] Raine, A. , Lencz, T. , Bihrle, S. , LaCasse, L. , & Colletti, P. (2000). Reduced prefrontal gray matter volume and reduced autonomic activity in antisocial personality disorder. Archives of General Psychiatry, 57(2), 119–127; discussion 128–119. 10.1001/archpsyc.57.2.119 10665614

[hbm25225-bib-0072] Raine, A. , Yang, Y. , Narr, K. L. , & Toga, A. W. (2011). Sex differences in orbitofrontal gray as a partial explanation for sex differences in antisocial personality. Molecular Psychiatry, 16(2), 227–236. 10.1038/mp.2009.136 20029391 PMC3008752

[hbm25225-bib-0073] Rashid, B. , Arbabshirani, M. R. , Damaraju, E. , Cetin, M. S. , Miller, R. , Pearlson, G. D. , & Calhoun, V. D. (2016). Classification of schizophrenia and bipolar patients using static and dynamic resting‐state fMRI brain connectivity. NeuroImage, 134, 645–657. 10.1016/j.neuroimage.2016.04.051 27118088 PMC4912868

[hbm25225-bib-0074] Rashid, B. , Damaraju, E. , Pearlson, G. D. , & Calhoun, V. D. (2014). Dynamic connectivity states estimated from resting fMRI identify differences among schizophrenia, bipolar disorder, and healthy control subjects. Frontiers in Human Neuroscience, 8, 897 10.3389/Fnhum.2014.00897 25426048 PMC4224100

[hbm25225-bib-0075] Reddy, P. G. , Mattar, M. G. , Murphy, A. C. , Wymbs, N. F. , Grafton, S. T. , Satterthwaite, T. D. , & Bassett, D. S. (2018). Brain state flexibility accompanies motor‐skill acquisition. NeuroImage, 171, 135–147. 10.1016/j.neuroimage.2017.12.093 29309897 PMC5857429

[hbm25225-bib-0076] Sadaghiani, S. , & D'Esposito, M. (2015). Functional characterization of the cingulo‐opercular network in the maintenance of tonic alertness. Cerebral Cortex, 25(9), 2763–2773. 10.1093/cercor/bhu072 24770711 PMC4537431

[hbm25225-bib-0077] Sanfratello, L. , Houck, J. , & Calhoun, V. D. (2019). Dynamic functional network connectivity in schizophrenia with MEG and fMRI, do different time scales tell a different story? Brain Connectivity, 9(3), 10.1089/brain.2018.0608 PMC6479258 30632385

[hbm25225-bib-0078] Scheinost, D. , Noble, S. , Horien, C. , Greene, A. S. , Lake, E. M. , Salehi, M. , … Constable, R. T. (2019). Ten simple rules for predictive modeling of individual differences in neuroimaging. NeuroImage, 193, 35–45. 10.1016/j.neuroimage.2019.02.057 30831310 PMC6521850

[hbm25225-bib-0079] Schultz, D. H. , & Cole, M. W. (2016). Integrated brain network architecture supports cognitive task performance. Neuron, 92(2), 278–279. 10.1016/j.neuron.2016.10.004 27764661

[hbm25225-bib-0080] Shen, H. , Li, Z. , Qin, J. , Liu, Q. , Wang, L. , Zeng, L. L. , … Hu, D. (2016). Changes in functional connectivity dynamics associated with vigilance network in taxi drivers. NeuroImage, 124(Pt A), 367–378. 10.1016/j.neuroimage.2015.09.010 26363345

[hbm25225-bib-0081] Shen, H. , Wang, L. , Liu, Y. , & Hu, D. (2010). Discriminative analysis of resting‐state functional connectivity patterns of schizophrenia using low dimensional embedding of fMRI. NeuroImage, 49(4), 3110–3121. 10.1016/j.neuroimage.2009.11.011 19931396

[hbm25225-bib-0083] Shirer, W. R. , Ryali, S. , Rykhlevskaia, E. , Menon, V. , & Greicius, M. D. (2012). Decoding subject‐driven cognitive states with whole‐brain connectivity patterns. Cerebral Cortex, 22(1), 158–165. 10.1093/cercor/bhr099 21616982 PMC3236795

[hbm25225-bib-0084] Spreng, R. N. , Mar, R. A. , & Kim, A. S. (2009). The common neural basis of autobiographical memory, prospection, navigation, theory of mind, and the default mode: A quantitative meta‐analysis. Journal of Cognitive Neuroscience, 21(3), 489–510. 10.1162/jocn.2008.21029 18510452

[hbm25225-bib-0085] Spreng, R. N. , Sepulcre, J. , Turner, G. R. , Stevens, W. D. , & Schacter, D. L. (2013). Intrinsic architecture underlying the relations among the default, dorsal attention, and frontoparietal control networks of the human brain. Journal of Cognitive Neuroscience, 25(1), 74–86. 10.1162/Jocn_a_00281 22905821 PMC3816715

[hbm25225-bib-0086] Su, J. , Shen, H. , Zeng, L.‐L. , Qin, J. , Liu, Z. , & Hu, D. (2016). Heredity characteristics of schizophrenia shown by dynamic functional connectivity analysis of resting‐state functional MRI scans of unaffected siblings. Neuroreport, 27(11), 843–848. 10.1097/WNR.0000000000000622 27295028

[hbm25225-bib-0087] Suk, H. I. , Wee, C. Y. , Lee, S. W. , & Shen, D. (2016). State‐space model with deep learning for functional dynamics estimation in resting‐state fMRI. NeuroImage, 129, 292–307. 10.1016/j.neuroimage.2016.01.005 26774612 PMC5437848

[hbm25225-bib-0088] Sundram, F. , Deeley, Q. , Sarkar, S. , Daly, E. , Latham, R. , Craig, M. , … Barker, G. J. (2012). White matter microstructural abnormalities in the frontal lobe of adults with antisocial personality disorder. Cortex, 48(2), 216–229. 10.1016/j.cortex.2011.06.005 21777912

[hbm25225-bib-0089] Syngelaki, E. M. , Moore, S. C. , Savage, J. C. , Fairchild, G. , & Van Goozen, S. H. M. (2009). Executive functioning and risky decision making in young male offenders. Criminal Justice and Behavior, 36(11), 1213–1227. 10.1177/0093854809343095

[hbm25225-bib-0090] Tanaka‐Matsumi, J. , & Kameoka, V. A. (1986). Reliabilities and concurrent validities of popular self‐report measures of depression, anxiety, and social desirability. Journal of Consulting and Clinical Psychology, 54(3), 328–333. 10.1037//0022-006x.54.3.328 3722561

[hbm25225-bib-0091] Tang, Y. , Jiang, W. , Liao, J. , Wang, W. , & Luo, A. (2014). Identifying individuals with antisocial personality disorder using resting‐state fMRI (vol 8, e60652, 2013). PLoS One, 9(4), e96962 10.1371/journal.pone.0096962 PMC3625191 23593272

[hbm25225-bib-0092] Thompson, G. J. , Magnuson, M. E. , Merritt, M. D. , Schwarb, H. , Pan, W. J. , McKinley, A. , … Keilholz, S. D. (2013). Short‐time windows of correlation between large‐scale functional brain networks predict vigilance intraindividually and interindividually. Human Brain Mapping, 34(12), 3280–3298. 10.1002/hbm.22140 22736565 PMC6870033

[hbm25225-bib-0093] Tomassini, V. , d'Ambrosio, A. , Petsas, N. , Wise, R. G. , Sbardella, E. , Allen, M. , … Carnì, M. (2016). The effect of inflammation and its reduction on brain plasticity in multiple sclerosis: MRI evidence. Human Brain Mapping, 37(7), 2431–2445. 10.1002/hbm.23184 26991559 PMC5069650

[hbm25225-bib-0094] Valk, S. L. , Bernhardt, B. C. , Trautwein, F.‐M. , Böckler, A. , Kanske, P. , Guizard, N. , … Singer, T. (2017). Structural plasticity of the social brain: Differential change after socio‐affective and cognitive mental training. Science Advances, 3(10), e1700489 10.1126/sciadv.1700489 28983507 PMC5627980

[hbm25225-bib-0095] Wang, C. H. , Ong, J. L. , Patanaik, A. , Zhou, J. , & Chee, M. W. L. (2016). Spontaneous eyelid closures link vigilance fluctuation with fMRI dynamic connectivity states. Proceedings of the National Academy of Sciences of the United States of America, 113(34), 9653–9658. 10.1073/pnas.1523980113 27512040 PMC5003283

[hbm25225-bib-0096] Wang, M. C. , Deng, Q. W. , Armour, C. , Bi, X. Y. , & Zeng, H. (2015). The psychometric properties and factor structure of the antisocial process screening device self‐report version in Chinese adolescents. Journal of Psychopathology and Behavioral Assessment, 37(4), 553–562. 10.1007/s10862-015-9486-x

[hbm25225-bib-0097] Wilson, R. S. , Mayhew, S. D. , Rollings, D. T. , Goldstone, A. , Przezdzik, I. , Arvanitis, T. N. , & Bagshaw, A. P. (2015). Influence of epoch length on measurement of dynamic functional connectivity in wakefulness and behavioural validation in sleep. NeuroImage, 112, 169–179. 10.1016/j.neuroimage.2015.02.061 25765256

[hbm25225-bib-0098] Wu, X. , Li, Q. , Yu, X. , Chen, K. , Fleisher, A. S. , Guo, X. , … Li, R. (2016). A triple network connectivity study of large‐scale brain Systems in Cognitively Normal APOE4 carriers. Frontiers in Aging Neuroscience, 8, 231 10.3389/fnagi.2016.00231 27733827 PMC5039208

[hbm25225-bib-0109] Wu, G. , Qi, F. , & Shen, D. (2006). Learning‐based deformable registration of MR brain images. IEEE Trans Med Imaging, 25(9), 1145–1157. 10.1109/TMI.2006.879320 16967800

[hbm25225-bib-0099] Xie, H. , Zheng, C. Y. , Handwerker, D. A. , Bandettini, P. A. , Calhoun, V. D. , Mitra, S. , & Gonzalez‐Castillo, J. (2018). Efficacy of different dynamic functional connectivity methods to capture cognitively relevant information. NeuroImage, 188, 502–514. 10.1016/j.neuroimage.2018.12.037 30576850 PMC6401299

[hbm25225-bib-0100] Yan, C. G. , Craddock, R. C. , Zuo, X. N. , Zang, Y. F. , & Milham, M. P. (2013). Standardizing the intrinsic brain: Towards robust measurement of inter‐individual variation in 1000 functional connectomes. NeuroImage, 80, 246–262. 10.1016/j.neuroimage.2013.04.081 23631983 PMC4074397

[hbm25225-bib-0101] Yan, W. , Zhang, H. , Sui, J. , & Shen, D. (2018). Deep chronnectome learning via full bidirectional long short‐term memory networks for MCI diagnosis. International Conference on MICCA, 2018, 249–257. 10.1007/978-3-030-00931-1_29 PMC6553484 31179447

[hbm25225-bib-0103] Yoder, K. J. , Harenski, C. , Kiehl, K. A. , & Decety, J. (2015). Neural networks underlying implicit and explicit moral evaluations in psychopathy. Translational Psychiatry, 5, e625 10.1038/tp.2015.117 26305476 PMC4564570

[hbm25225-bib-0104] Zeng, L. L. , Wang, D. , Fox, M. D. , Sabuncu, M. , Hu, D. , Ge, M. , … Liu, H. (2014). Neurobiological basis of head motion in brain imaging. Proceedings of the National Academy of Sciences of the United States of America, 111(16), 6058–6062. 10.1073/pnas.1317424111 24711399 PMC4000812

[hbm25225-bib-0105] Zeng, L. L. , Wang, H. , Hu, P. , Yang, B. , Pu, W. , Shen, H. , … Tan, Q. (2018). Multi‐site diagnostic classification of schizophrenia using discriminant deep learning with functional connectivity MRI. eBioMedicine, 30, 74–85. 10.1016/j.ebiom.2018.03.017 29622496 PMC5952341

[hbm25225-bib-0106] Zhang, H. , Chen, X. , Zhang, Y. , & Shen, D. (2017). Test‐retest reliability of “high‐order” functional connectivity in young healthy adults. Frontiers in Neuroscience, 11, 439 10.3389/fnins.2017.00439 28824362 PMC5539178

[hbm25225-bib-0107] Zhou, Z. , Chen, X. , Zhang, Y. , Hu, D. , Qiao, L. , Yu, R. , … Shen, D. (2020). A toolbox for brain network construction and classification (BrainNetClass). Human Brain Mapping, 41(10), 2808–2826. 10.1002/hbm.24979 32163221 PMC7294070

[hbm25225-bib-0108] Zung, W. W. (1971). A rating instrument for anxiety disorders. Psychosomatics, 12(6), 371–379. 10.1016/S0033-3182(71)71479-0 5172928

