# Supplementary material for: Dynamic neural circuit disruptions associated with antisocial behaviors
Source: Hum Brain Mapp. 2020 Oct 16;42(2):329–44. doi: 10.1002/hbm.25225 (PMC7776000; doi:10.1002/hbm.25225)
Supplement: Supplementary file 1 — FIGURE S1 The distribution of APSD scores. APSD: Antisocial Process Screening Device FIGURE S2. (a) The distribution of SAS scores; (b) Scatter plot between the model‐generated and the observed SAS scores for the integrated model. SAS: Self‐rating Anxiety Scale TABLE S1. Network‐wise ASB Score Association FIGURE S3. Scatter plot between the model‐generated and observed ASB scores with the dFC between DMN and FPN. TABLE S2. The Pearson correlation between the 103 contributive dFC‐ALFF features and the extent of head motion [file HBM-42-329-s001.docx]

**Supplemental Document**

**Dynamic neural circuit disruptions associated with antisocial behaviors**

Weixiong Jiang ^a^, Han Zhang ^a*^, Ling-Li Zeng ^b^, Hui Shen ^b^, Jian Qin ^b^, Kim-Han Thung ^a^, Pew-Thian Yap ^a^, Huasheng Liu ^c^, Dewen Hu ^b^, Wei Wang ^c*^, Dinggang Shen ^a,d*^

**ASB Score Distribution**

In this study, we used Antisocial Process Screening Device (APSD) to measure the severity of individual antisocial behavior (ASB). The APSD scores of all the subjects ranged from 6 to 29, with a mean of 16.69 and a median of 17 (Figure S1).

Figure S1. The distribution of APSD scores. APSD: Antisocial Process Screening Device.

**Investigation of individual emotional status and control correlations**

In this experiment, we screened individual emotional status using Self-rating Anxiety Scale (SAS) (Zung, 1971), which has good psychometric credentials (Tanaka-Matsumi & Kameoka, 1986). The SAS has been extensively used in research, especially in medical disciplines (Dunstan & Scott, 2018).

As control correlations, we used SAS scores as targets feeding into the associative model instead of the APSD scores. Our subjects had the SAS scores ranging from 24 to 57, with the mean 37.41 and median 36 (Figure S2A). The predictive results showed no significant association between the dFC-ALFF and SAS scores (*r* = -0.0691, *p* = 0.4631) (Figure S2B).


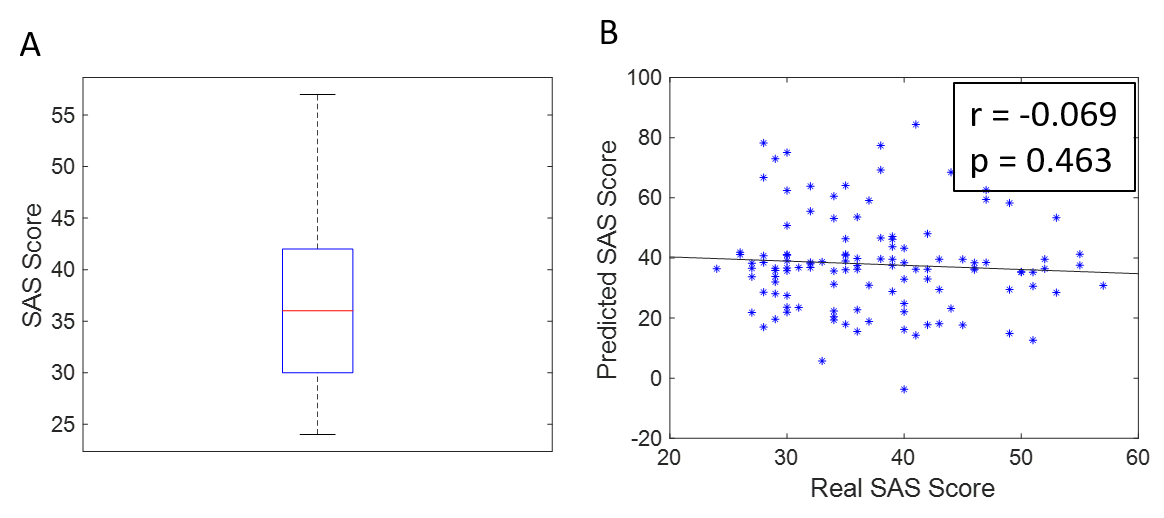


Figure S2. (A) The distribution of SAS scores; (B) Scatter plot between the model-generated and the observed SAS scores for the integrated model. SAS: Self-rating Anxiety Scale.

**Performance of Network-specific ASB Score Association**

As shown in Table S1, none of the six networks showed any significant association with ASB scores (*p* > 0.05) when only intra-network links of each network was considered. When we used pairwise inter-network connections to associate with ASB scores, the dFC between CON and FPN presented a good associative ability (*r* = 0.23, *p* < 0.05, FDR corrected), while the dFC between DMN and FPN showed an even better associative ability (*r* = 0.41, *p* < 0.05, FDR corrected, see Figure S3 as below).

Table S1. Network-wise ASB Score Association

| Network | *r* | *p* |
| --- | --- | --- |
| **Intra-network** |  |  |
| CON | 0.0190 | 0.8401 |
| FPN | 0.1165 | 0.2149 |
| DMN | 0.0555 | 0.5556 |
| SMN | -0.0002 | 0.9985 |
| **Inter-network** | | |
| CON-DMN | 0.1572 | 0.0934 |
| CON-FPN | 0.2325 | 0.0124* |
| CON-SMN | -0.1759 | 0.0601 |
| DMN-FPN | 0.4133 | 4.41×10^-6^* |
| DMN-SMN | 0.0348 | 0.7120 |
| FPN-SMN | -0.0725 | 0.4415 |
| *: significant correlation, FDR corrected.  CON: cingulo-opercular network; DMN: default mode network; FPN: fronto-parietal network; SMN: sensorimotor network. | | |


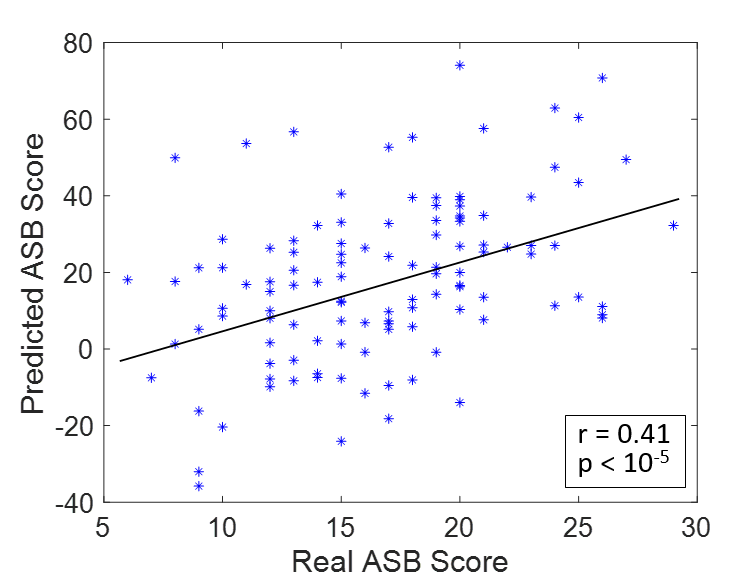


Figure S3. Scatter plot between the model-generated and observed ASB scores with the dFC between DMN and FPN.

**Head motion effect on the identified ASB-related dFC**

Table S2. The Pearson correlation between the 103 contributive dFC-ALFF features and the extent of head motion

| FC | *r* | *p* | FC | *r* | *p* | FC | *r* | *p* |
| --- | --- | --- | --- | --- | --- | --- | --- | --- |
| 1 | -0.1020 | 0.2783 | 36 | 0.0295 | 0.7545 | 71 | -0.0564 | 0.5497 |
| 2 | -0.0687 | 0.4655 | 37 | 0.0787 | 0.4034 | 72 | -0.1400 | 0.1357 |
| 3 | -0.0510 | 0.5884 | 38 | -0.1503 | 0.1089 | 73 | -0.0215 | 0.8195 |
| 4 | -0.0506 | 0.5910 | 39 | -0.0198 | 0.8340 | 74 | 0.1719 | 0.0662 |
| 5 | -0.0845 | 0.3691 | 40 | -0.1187 | 0.2063 | 75 | 0.0530 | 0.5739 |
| 6 | -0.0314 | 0.7392 | 41 | 0.0047 | 0.9606 | 76 | 0.0211 | 0.8227 |
| 7 | 0.0140 | 0.8823 | 42 | 0.1864 | 0.0460* | 77 | -0.0150 | 0.8732 |
| 8 | -0.0363 | 0.7004 | 43 | 0.0135 | 0.8865 | 78 | 0.0387 | 0.6817 |
| 9 | 0.0288 | 0.7598 | 44 | 0.0074 | 0.9378 | 79 | -0.0161 | 0.8644 |
| 10 | 0.0511 | 0.5879 | 45 | -0.1152 | 0.2203 | 80 | -0.0023 | 0.9805 |
| 11 | 0.1352 | 0.1497 | 46 | -0.1707 | 0.0682 | 81 | 0.0376 | 0.6899 |
| 12 | -0.1295 | 0.1678 | 47 | -0.1784 | 0.0565 | 82 | -0.0173 | 0.8541 |
| 13 | -0.0279 | 0.7672 | 48 | -0.0610 | 0.5176 | 83 | -0.0175 | 0.8529 |
| 14 | -0.1081 | 0.2501 | 49 | 0.0434 | 0.6452 | 84 | -0.0577 | 0.5399 |
| 15 | 0.0693 | 0.4615 | 50 | -0.1320 | 0.1596 | 85 | 0.1395 | 0.1370 |
| 16 | 0.1199 | 0.2018 | 51 | 0.0116 | 0.9020 | 86 | -0.0299 | 0.7507 |
| 17 | 0.1554 | 0.0973 | 52 | -0.0193 | 0.8381 | 87 | 0.0178 | 0.8506 |
| 18 | 0.0010 | 0.9917 | 53 | 0.1250 | 0.1831 | 88 | -0.0304 | 0.7474 |
| 19 | -0.1276 | 0.1742 | 54 | 0.0930 | 0.3228 | 89 | 0.0289 | 0.7588 |
| 20 | 0.0342 | 0.7170 | 55 | -0.0355 | 0.7063 | 90 | 0.0817 | 0.3856 |
| 21 | -0.0502 | 0.5942 | 56 | 0.0759 | 0.4202 | 91 | 0.0253 | 0.7887 |
| 22 | -0.0320 | 0.7344 | 57 | 0.0581 | 0.5374 | 92 | 0.0045 | 0.9619 |
| 23 | 0.0016 | 0.9867 | 58 | 0.0912 | 0.3324 | 93 | -0.2131 | 0.0222* |
| 24 | 0.1781 | 0.0569 | 59 | 0.0816 | 0.3857 | 94 | -0.0034 | 0.9709 |
| 25 | 0.0072 | 0.9391 | 60 | 0.1326 | 0.1577 | 95 | 0.0353 | 0.7083 |
| 26 | -0.0755 | 0.4226 | 61 | 0.0277 | 0.7690 | 96 | 0.0468 | 0.6196 |
| 27 | 0.1140 | 0.2250 | 62 | 0.1793 | 0.0552 | 97 | 0.0202 | 0.8301 |
| 28 | 0.0756 | 0.4219 | 63 | -0.0013 | 0.9887 | 98 | -0.0863 | 0.3590 |
| 29 | 0.0306 | 0.7454 | 64 | -0.1242 | 0.1861 | 99 | 0.0045 | 0.9623 |
| 30 | 0.2754 | 0.0029* | 65 | -0.1103 | 0.2408 | 100 | -0.0847 | 0.3682 |
| 31 | -0.0957 | 0.3092 | 66 | -0.0716 | 0.4469 | 101 | 0.0679 | 0.4711 |
| 32 | -0.0604 | 0.5216 | 67 | -0.0935 | 0.3204 | 102 | 0.1067 | 0.2565 |
| 33 | -0.0741 | 0.4315 | 68 | 0.1225 | 0.1921 | 103 | 0.0331 | 0.7252 |
| 34 | -0.0765 | 0.4164 | 69 | 0.0627 | 0.5054 |  |  |  |
| 35 | -0.0428 | 0.6498 | 70 | -0.0675 | 0.4738 |  |  |  |

*: p < 0.05, uncorrected

**References**

Dunstan, D. A., & Scott, N. (2018). Assigning clinical significance and symptom severity using the Zung scales: levels of misclassification arising from confusion between index and raw scores. *Depress Res Treat, 2018*. doi: 10.1155/2018/9250972

Power, J. D., Barnes, K. A., Snyder, A. Z., Schlaggar, B. L., & Petersen, S. E. (2012). Spurious but systematic correlations in functional connectivity MRI networks arise from subject motion. *Neuroimage, 59*(3), 2142-2154. doi:10.1016/j.neuroimage.2011.10.018

Tanaka-Matsumi, J., & Kameoka, V. A. (1986). Reliabilities and concurrent validities of popular self-report measures of depression, anxiety, and social desirability. *J Consult Clin Psychol, 54*(3), 328-333. doi:10.1037//0022-006x.54.3.328

Zeng, L. L., Wang, D., Fox, M. D., Sabuncu, M., Hu, D., Ge, M., . . . Liu, H. (2014). Neurobiological basis of head motion in brain imaging. *Proc Natl Acad Sci U S A, 111*(16), 6058-6062. doi:10.1073/pnas.1317424111

Zung, W. W. (1971). A rating instrument for anxiety disorders. *Psychosomatics, 12*(6), 371-379. doi:10.1016/S0033-3182(71)71479-0
